# Computer-aided craniofacial superimposition validation study: the identification of the leaders and participants of the Polish-Lithuanian January Uprising (1863–1864)

**DOI:** 10.1007/s00414-022-02929-4

**Published:** 2022-12-15

**Authors:** Rubén Martos, Rosario Guerra, Fernando Navarro, Michela Peruch, Kevin Neuwirth, Andrea Valsecchi, Rimantas Jankauskas, Oscar Ibáñez

**Affiliations:** 1 Panacea Cooperative Research S. Coop, Ponferrada, Spain; 2 Physical Anthropology Lab, Department of Legal Medicine, Toxicology and Physical Anthropology, University of Granada, Granada, Spain; 3 Department of Medicine and Surgery, University of Trieste, Trieste, Italy; 4 Institute for Prehistory, Early History and Medieval Archaeology, University of Tübingen, Tübingen, Germany; 5 Andalusian Research Institute in Data Science and Computational Intelligence, University of Granada, Granada, Spain; 6 Department of Anatomy, Histology and Anthropology, Vilnius University, Vilnius, Lithuania; 7 Faculty of Computer Science, CITIC, University of A Coruna, 15071 La Coruña, Spain

**Keywords:** Skull-face overlay, Craniofacial superimposition, Craniofacial identification, Forensic Anthropology, Artificial Intelligence

## Abstract

In 2017, a series of human remains corresponding to the executed leaders of the “January Uprising” of 1863–1864 were uncovered at the Upper Castle of Vilnius (Lithuania). During the archeological excavations, 14 inhumation pits with the human remains of 21 individuals were found at the site. The subsequent identification process was carried out, including the analysis and cross-comparison of post-mortem data obtained in situ and in the lab with ante-mortem data obtained from historical archives. In parallel, three anthropologists with diverse backgrounds in craniofacial identification and two students without previous experience attempted to identify 11 of these 21 individuals using the craniofacial superimposition technique. To do this, the five participants had access to 18 3D scanned skulls and 14 photographs of 11 different candidates. The participants faced a cross-comparison problem involving 252 skull-face overlay scenarios. The methodology follows the main agreements of the European project MEPROCS and uses the software Skeleton-ID™. Based on MEPROCS standard, a final decision was provided within a scale, assigning a value in terms of strong, moderate, or limited support to the claim that the skull and the facial image belonged (or not) to the same person for each case. The problem of binary classification, positive/negative, with an identification rate for each participant was revealed. The results obtained in this study make the authors think that both the quality of the materials used and the previous experience of the analyst play a fundamental role when reaching conclusions using the CFS technique.

**Supplementary Information:**

The online version contains supplementary material available at 10.1007/s00414-022-02929-4.

## Introduction

In the beginning of 2017, archeological excavations carried out by the Lithuanian National Museum in the Upper Castle on Gediminas Hill, in the capital city of Vilnius, uncovered a series of human remains. The archeological artifacts found at the burial place confirmed that the clandestine inhumation pits could contain the remains of military leaders executed during the Polish-Lithuanian rebellion against the Russian Empire in the nineteenth century, known as the “January Uprising” (1863–1864).

Systematic archeological excavations, alongside the relevant anthropological and historical investigations, were performed, uncovering a total of 20 human remains buried in 14 inhumation pits. To identify these remains, for a later state burial, they proceeded to perform the analysis and cross-comparison of the post-mortem (PM) data (estimated age, sex, stature, pathological and traumatological conditions) with the ante-mortem (AM) data (age, date, execution method, and other data from historical archives). A genealogic search for live and deceased relatives to carry out identification through next generation DNA sequencing took place. In November 2019, after the identification was successfully completed, a state funeral was celebrated.

Apart from the aforementioned identification methods, thanks to the availability of photographs belonging to 11 individuals, identification through craniofacial superimposition (CFS) was carried out. This technique involves the direct comparison of the image or 3D model of an unknown skull with the photograph of a known person, analyzing the morphological correspondences to determine, with a reasonable degree of confidence, whether they belong to the same person or not [1–3].

Even though the CFS technique has been in use for over a century [4], it is still controversial among the scientific community [5]. The scarcity of studies and their limitations [6] lead to contradictory information regarding its reliability. Instead of following a consistent methodology, each expert tends to apply their own approach to the problem, based on the available equipment and their own previous knowledge on craniofacial anatomy. Within the context of the European project “New Methodologies and Protocols of forensic identification by Craniofacial Superimposition” (MEPROCS) [7], one of the main goals was to propose a common framework, avoiding individual approaches that could skew the process, to allow the application of the CFS technique in real-life forensic identification scenarios. This publication became the first CFS standard, compiling 17 best practice items together with the main sources of error and uncertainty in CFS. Additionally, it includes a document listing all the technical requirements and desirable characteristics that video and computer-aided CFS systems should possess for the correct application of the technique.

The methodological agreements reached at the MEPROCS consortium have been followed by five different participants, using Skeleton-ID™ software solution [8]. This is the first and only software tool to include all the advised requirements and features according to the standard. In addition, Skeleton-ID™ includes an Artificial Intelligence algorithm able to superimpose a 3D skull model and a facial photograph in less than a second from a set of homologous cranial and facial landmarks and their corresponding soft tissue depth measures [9]. Therefore, the current study represents the first blind validation of the application of the methodological framework defined by MEPROCS using Skeleton-ID™ software in a real-life identification scenario. The CFS technique has been applied to every possible AM-PM match independently and with no previous access to the prior identifications carried out by other methods.

## Previous validation studies

Of the various thanatological identification techniques, CFS is considered a controversial technique within the scientific community. Some authors classify it as a “useful” and “powerful” technique for positive identification. However, many others believe that CFS is more suitable for exclusion of individuals or as a source of corroborative evidence. In between, some authors [1, 2] claim that it is not possible to make firm statements about the overall reliability of CFS methods due to the small number of published studies, the small samples used, and the significant number of limitations of these studies (as will be discussed below). Moreover, in no case have they been replicated.

Among all the studies published to date, there are three groups that can be distinguished. The first studies, in which video-superimposition systems were used, reported high reliability, 100% in three [3–5] of the five works published [3–7]. However, two of them [3, 5] are approaches not applicable in practice because they use a radiograph of the living subject and achieve perfect skull-face overlays (SFOs). In the third of these [4], the approach is totally different and instead of measurements, 39 morphological criteria are analyzed.

The two works that do not report 100% accuracy [6, 7] determine that with 12 and 13 criteria analyzed as consistent respectively, a positive conclusion can be reached. Their studies conclude that the technique is much more reliable when applied to two or more photos of the same subject, where they report 0.6% false positives and 96% true positives, respectively.

Regarding the samples employed, in [4, 7] the authors analyzed 52 and 30 cases previously identified in their respective laboratories, without cross-comparison. Austin and Maples [6] performed cross-comparison of 3 skulls and 97 subjects with frontal and lateral photos, with all cases being negative. In the work of Ricci et al. [5], a cross-comparison was made with 14 subjects, i.e., 196 CFS problems. Finally, the work of Chai et al. [3] is the most extensive, as it involved 224 volunteers who underwent 27 radiographs at different angles with the aim of studying a total of 52 indices and criteria in the resulting 6048 radiographs. In addition, although they give little detail, they claim to have carried out 10,000 cross-comparisons of 10 skulls with 1000 facial X-rays.

A second group of more recent studies carried out a number of cross-comparisons ranging from 50 by Dr. Cristina Cattaneo’s group (Italy) [8] to 400 in studies by Dr. Maryna Steyn’s group in South Africa [9] and Dr. Caroline Wilkinson’s group in the UK [10]. In all cases, 3D skull models and generic image processing software were used for the SFOs and their evaluations.

In the work of Gordon et al. [9] and Gaudio et al. [8], approaches based on the use of landmarks, morphological criteria, and a combination of both were studied, whereas in the case of Wilkinson’s study [10] only a morphological approach was followed.

In contrast to the previous group of studies, the results are much worse. False positive rates range from 8 to 20% and true positives from 50 to 85%. It is true that in these three studies only one photograph was used.

Finally, there are three studies which, although not aimed at studying the reliability of the technique, are closely related to this subject. The first two are part of the European MEPROCS project, the aim of which was to establish international guidelines in CFS. The first [11] involved 26 experts from 17 institutions who worked with their own means and methodologies on the same set of identification cases. The results were very disparate and served to identify good and bad practices, as well as to analyze technical and methodological aspects. The second study [12] confirmed that the reliability of the technique increases with the use of the MEPROCS framework. Finally, the third study [13] included in this last group aimed to take the first steps to design a system to support decision-making and automatic evaluation of morphological correspondences. Despite being a very partial system, it achieved 90% of correct decisions when dealing, in a fully automatic way, with the identification scenarios proposed in the first of the studies. This percentage was only surpassed by the best of the 26 participants. This paper proposes an alternative use to the identification-exclusion discussion: the automatic filtering of cases in multiple comparison scenarios, so that forensic experts only have to examine a small number of cases.

However, as discussed at the beginning of this section, all the studies conducted are fraught with limitations:

- In some cases, the experimental design does not represent a realistic scenario as the variables are artificially and unrealistically controlled.
- The size and composition of the sample is insufficient to draw statistically significant conclusions. The number of negative cases far exceeds the number of positive cases. No study exceeds 20 positive cases, in which only one photo is used. At best, the number of cross-comparisons is 400.
- There are publications where only negative or positive cases have been used, but not both types in the same study.
- SFOs were obtained after a subjective trial-and-error procedure in almost all studies. Where not, the automatic method is not entirely accurate and the authors themselves point out that there is much room for improvement.
- There are a number of limitations that undoubtedly affect the quality of the SFO carried out and the consequent decision-making. Dispersion in the marking of cephalometric and craniometric landmarks is not adequately considered and modeled. The software used lacks specific tools for more accurate marking.
- Along the same lines, software not designed for SFO is used, which does not model perspective and other camera parameters and does not allow visualization of the estimated soft tissue thickness.
- Furthermore, in some cases, significant errors were made, such as using previously cropped photographs, using photographs of cadavers (with the consequent change in morphology and soft tissue thickness), or the use of inaccurate and untextured 3D models.
- When assessing correspondence with a morphological approach the individual assessments are not provided and it is a subjective process. In landmark-based approaches there are no measurements or numerical approximations. There is a high expert and/or technology bias. There is an almost total absence of inter- and intra-observer error measurement.
- Virtually all studies cannot be replicated or quantitatively compared with each other. One of the main reasons is the absence of public and/or shared data.

## Materials and methods

The dataset used in this study was limited to the 3D skull models of 18 out of the 20 human remains found and 14 photographs belonging to 11 different candidates. To conduct a blind study, the available information about biological profile of the candidates, circumstantial evidence found (personal belongings such as a wedding ring with engraved initials of the subject’s name), or other information obtained from historical archives (dates and methods of execution, injuries) and identification using DNA sequencing, was not revealed to the participating researchers. Hence, due to the impossibility of undertaking a previous filtering, this identification task supposes a challenge involving a total of 252 (18 skull 3D models × 14 photographs) SFOs, pertaining to 198 CFS problems (18 skulls × 11 candidates).

The study was carried out independently by five participants (three anthropologists and two students), with different levels of experience and proficiency in the application of the technique, from three different institutions: University of Granada (Spain), University of Tübingen (Germany), and University of Trieste (Italy). Table 1 lists all the participants (numbered from 1 to 5) in the study with the corresponding academic background and level of CFS experience. Prior to the identification process, both graduate students (P1 and P2) were provided with extensive training material related to CFS.

**Table 1:** Participants of the study, their academic background, and their experience related to CFS

Participant number	Academic background	CFS experience
P1	Graduate student in Medicine	No previous experience with CFS
P2	Graduate student in Biology	No previous experience with CFS
P3	Post-graduate student in Physical and Forensic Anthropology	Short previous research experience and CFS-related training
P4	PhD in Physical and Forensic Anthropology	Broad experience with CFS real cases
P5	PhD in Physical and Forensic Anthropology	Broad experience with CFS real cases

With the aim of tackling this complex identification scenario, we used the Skeleton-ID™ software [14] developed by Panacea Cooperative Research. It includes the automatic algorithm POSEST-SFO [15] for the SFO task and it is the first software tool to meet all the requirements and technical features recommended by the MEPROCS consortium [16] for computer-aided application of the CFS technique. These recommendations and guidelines were compiled for the purpose of minimizing and avoiding the main sources of error and dealing with the uncertainty in the application of the technique.

A three consecutive stage process was followed, as described in the most recent CFS literature reviews [17, 18]:

- The acquisition and processing of the materials stage, meaning the 3D scanning of the skull and the digital scanning of AM facial photographs, as well as the subsequent cephalometric and craniometric landmarks location.
- The SFO stage, focused on achieving the most precise overlay of the skull 3D model over an AM facial photograph. This process is repeated for each available photograph of any candidate, obtaining different SFOs for each subject.
- The decision-making stage, in which the results of the different SFOs are evaluated. The identification decision is made by either judging the matching between the corresponding landmarks in the skull and the face, or by analyzing the morphological correlation between skull and face taking into consideration the values of the soft tissue depth measures and the consistency of any existing asymmetries.

### Stage 1: acquisition and preprocessing of the materials

For the purpose of obtaining three dimensional models of the 18 skulls, a structured-light 3D scanner (Go!SCAN 20™) was used. The task was carried out in Vilnius, following good practices established by MEPROCS, scanning the mandible and the rest of the skull separately. Then, for the articulation of the 3D models the automatic algorithm developed in [19] was used. It is important to mention that of the 18 scanned skulls, 17 were complete and preserved in good condition for CFS. Only one of the skulls was fragmented due to the impact of a projectile and therefore had to be reassembled before being scanned. Images of the scanned skulls can be found in the Online Resource 1. The 14 photographs of the candidates had been collected from different historical archives and subsequently digitized using an Epson Perfection v700 scanner, maintaining the original aspect ratio.

The landmarks used for identification were located in the face and skull, giving priority to those found in areas where soft tissue depth values are lower, less influenced by changes related to age, weight, or facial expression. A set of 18 cephalometric landmarks and their craniometric homologs [20] (Table 2) was selected, and subsequently marked on the 3D skull models and the AM facial photographs. With the purpose of meeting MEPROCS good practice recommendations, an anthropologist at Vilnius University was asked to locate and mark the craniometric landmarks on the physical skull before scanning, to later guide their location on the 3D models.

**Table 2:** List of cephalometric and homologous craniometric landmarks considered during the CFS process

Cephalometric landmark	Abv	Craniometric landmark	Abv
Vertex	v’	Vertex	v
Glabella	g’	Glabella	g
Nasion	n’	Nasion	n
Subnasale	sn’	Subspinale	ss
Labiale superius	ls’	Prosthion	pr
Labiale inferius	li’	Infradentale	id
Pogonion	pg’	Pogonion	pg
Gnathion	gn’	Gnathion	gn
Gonion L/R	go’	Gonion L/R	go
Alare L/R	al’	Alare L/R	al
Zygion L/R	zy’	Zygion L/R	zy
Endocanthion L/R	en’	Dacryon L/R	d
Exocanthion L/R	ex’	Ectoconchion L/R	ec

However, said markings were not followed due to several discrepancies in the location of certain landmarks according to Caple and Stephan [24], therefore new markings were carried out on the 3D models independently by the five participants involved in the study.

**Fig. 1:**
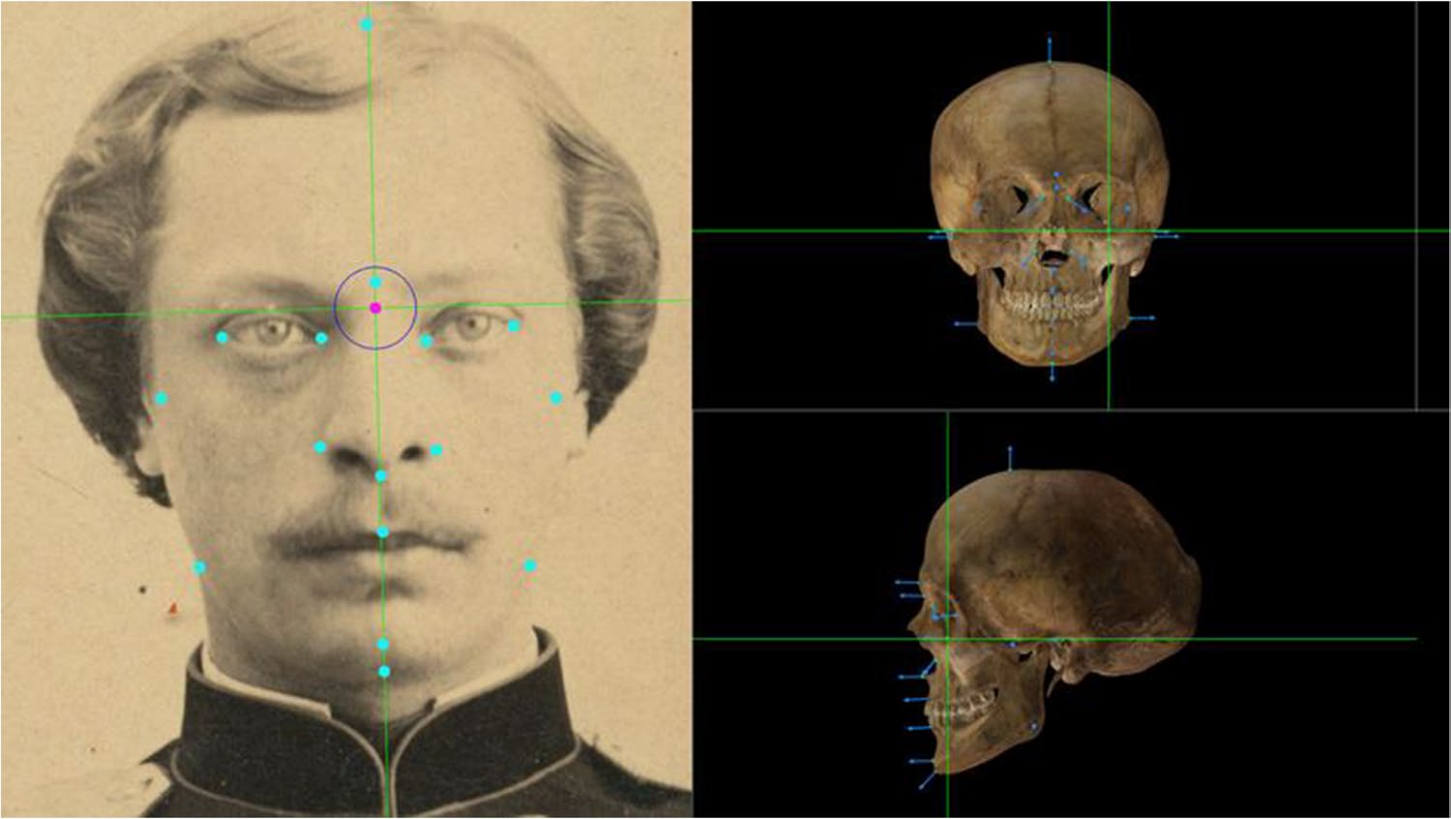
Cephalometric (left) and craniometric (right) landmark location using the tools provided by Skeleton-ID™

Skeleton-ID™ was used to load and display the photographs and skull 3D models. Afterwards, a set of tools specifically designed to reduce intra- and inter-observer cephalometric and craniometric landmark location errors [21] were employed (Fig. 1).

- Crosshair tool with 2D auxiliary transversal lines to enable more accuracy and facilitate the marking of cephalometric landmarks (on just one pixel) with relation to other regions or anatomical structures.
- Tool to establish the Frankfurt plane on the skull.
- Four-screen display of the different views of the skull used in anatomical descriptions (norma verticalis, norma occipitalis, norma basalis, norma frontalis, and norma lateralis) while positioned in the Frankfurt plane, facilitating the precise marking of the craniometric landmarks.
- Crosshair tool with 3D auxiliary transversal lines to enable accurate and time-efficient marking of the craniometric landmarks in relation to other landmarks or anatomical structures, allowing interaction with four simultaneous views to aid in unambiguously locating the most extremal point in a region of the 3D skull model surface.

### Stage 2: skull-face overlay

**Fig. 2:**
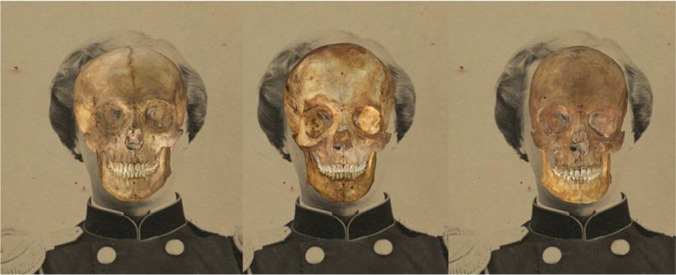
SFOs belonging to three different skulls over the same photograph, all obtained using the POSEST-SFO automatic algorithm

The 252 SFOs were obtained via the automatic algorithm POSEST-SFO [15] (Fig. 2), which itself uses the pinhole camera model, and in particular is able to calculate the mathematically optimal position, orientation, and focal length for the camera. This provides all the information to match the perspective of the photo, provided the landmarks have been located throughout the face. This method is extraordinarily fast, offering results in less than 78 ms. Afterwards, a manual refinement of every overlay that required it, was carried out (i.e., those cases that presented occlusions or blockages of the facial anatomy due to clothing, hair, shadows, etc.).

The soft tissue thickness average values were drawn from Stephan data for adult world population [22].

Skeleton-ID™ was used to apply the automatic SFO algorithm and for the subsequent refinement of the 3D skull model over the photograph, by means of the rotation, translation, and projection tools available in the software. The latter includes the possibility to adjust camera parameters as film size, focal distance, subject-to-camera distance, and the principal point [23].

The SFOs obtained in Skeleton-ID follow the pinhole camera model, which mathematically represents the essential behavior of any camera system [24, 25], including the early cameras available in the 1860s, and thus is considered suited for this study. The skull is displayed the same way it would appear in an actual photograph; in particular, perspective distortion is applied accordingly. The matching of the perspective was achieved by either the automatic SFO algorithm directly or through refinement by the analysts using an extensive trial-and-error process. The matching of the landmarks, the overall shape of the skull and face, and the consistency of corresponding anatomical features are considered while doing so. It is worth mentioning that Skeleton-ID offers a perspective adjustment tool, which allows the user to conveniently change the amount of perspective distortion. This is obtained using the so-called dolly zoom, i.e., moving the camera closer and while zooming out so that the skull is kept in the same apparent position and size.

### Stage 3: decision-making

To reach a final conclusion over the identification of each SFO, a set of morphological criteria was used to evaluate the anatomical consistency between the superimposed skull and the face. These criteria are grouped as follows: (1) evaluation of asymmetries; (2) correspondence between facial and bony curves; (3) soft tissue evaluation analyzing the relative position of cranial landmarks and their facial homologs while considering the soft tissue depth values; and (4) anatomical consistency through positional relationship of facial and skeletal structures. These examination criteria are based on previous works by Chai et al. [3], Austin and Maples [6], Yoshino et al. [7], or Jayaprakash et al. [4], which are compiled and defined in Ibáñez et al. [12], where the authors proposed a method based on the observation of morphological patterns.

The most representative criteria used for the evaluation of morphological correspondence in this study can be found in Table 3.

**Table 3:** Description of the most representative criteria used to assess the morphological correspondence

Consistency analysis of the facial and bony morphological curves or outlines
• The outline of the frontal bone of the skull follows the forehead outline of the facial photograph • The skull height is similar to the head height (allowing for explicable soft tissue depth variability and perceptual distortion caused by presence of hair) • The width of the cranium fills forehead area of the face • The width of the skull from menton to bregma fits within the face • The lateral line of the zygomatic bone matches the outline of the cheek • The chin outline is consistent with the mental outline • The arcus supraciliary follows the supraorbital margin
Anatomical consistency assessment by positional relationship
• The porion aligns just posterior to the tragus, slightly inferior to the crus of helix • Whitnall’s tubercle aligns with the ectocanthus on the horizontal plane and vertically the ectocanthus lies medial to the tubercle. The orbital width is consistent with the eye-slit width • The medial margin of the orbit aligns and superimposes with the endocanthion • The lower margin of piriform aperture matches the subnasale • The piriform aperture width and height lies within the borders of the nose
Consistency of the soft tissue thickness between corresponding landmarks
• Evaluation of the consistency of the facial soft tissue thickness considering distances between pairs of homologous landmarks located on the skull and the face. The soft tissue depth values are extracted from the soft tissue depth study uploaded and visually represented by colored 3D cones
Asymmetries evaluation
• Nasal area asymmetries • Zygomatic area asymmetries • Orbital area asymmetries

The criteria were visually assessed with aid of specific graphical tools, such as transparency and wipe, recommended by MEPROCS, and a visual representation of the soft tissue depth statistical information.

**Fig. 3:**
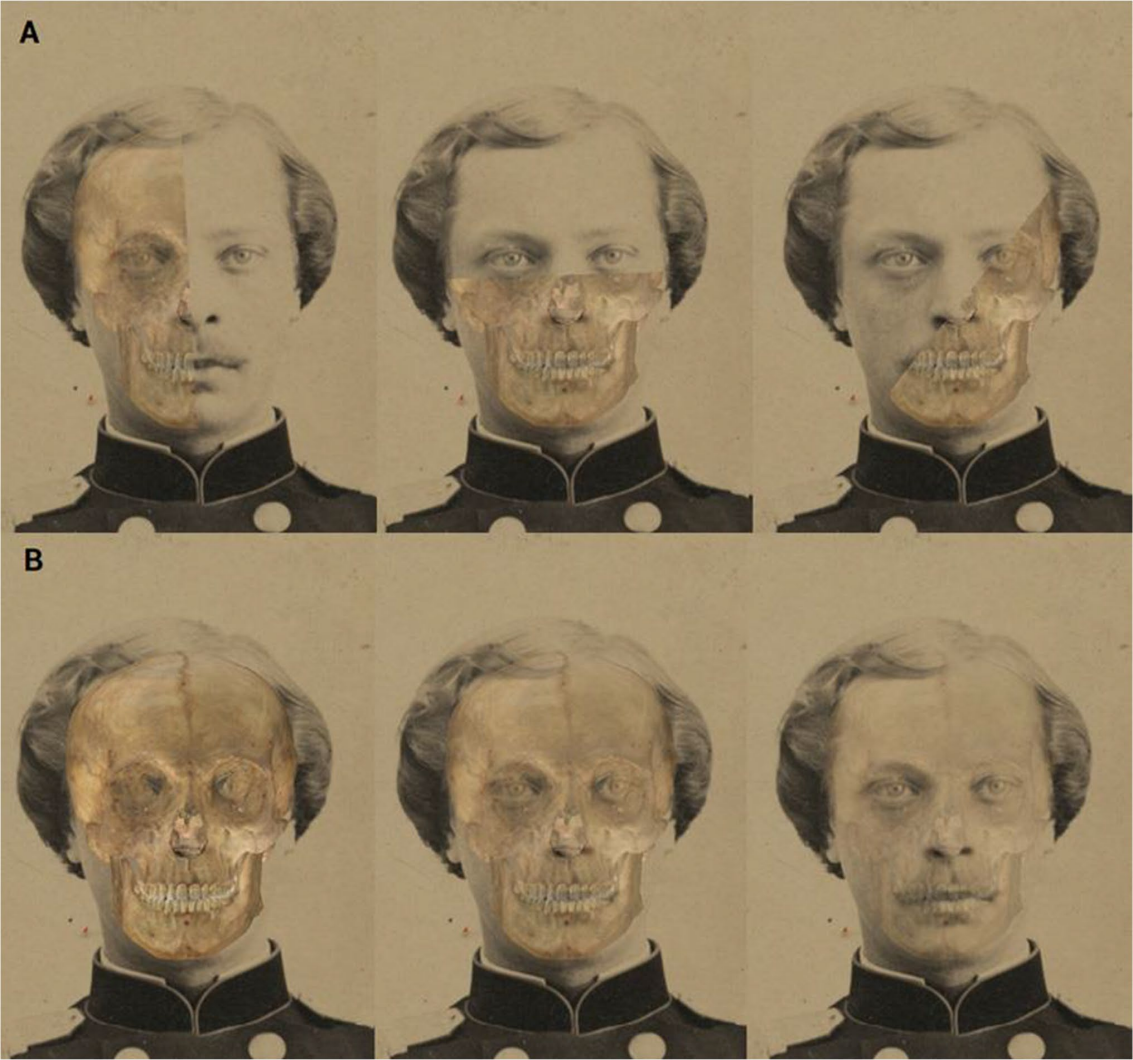
Evaluation of morphological correspondence of the skull and the face on a SFO using Skeleton-ID™. A Visual assessment using the wipe tool (vertical, horizontal, and diagonal). B Visual assessment using the transparency/opacity tool (75%, 50%, and 25%)

**Fig. 4:**
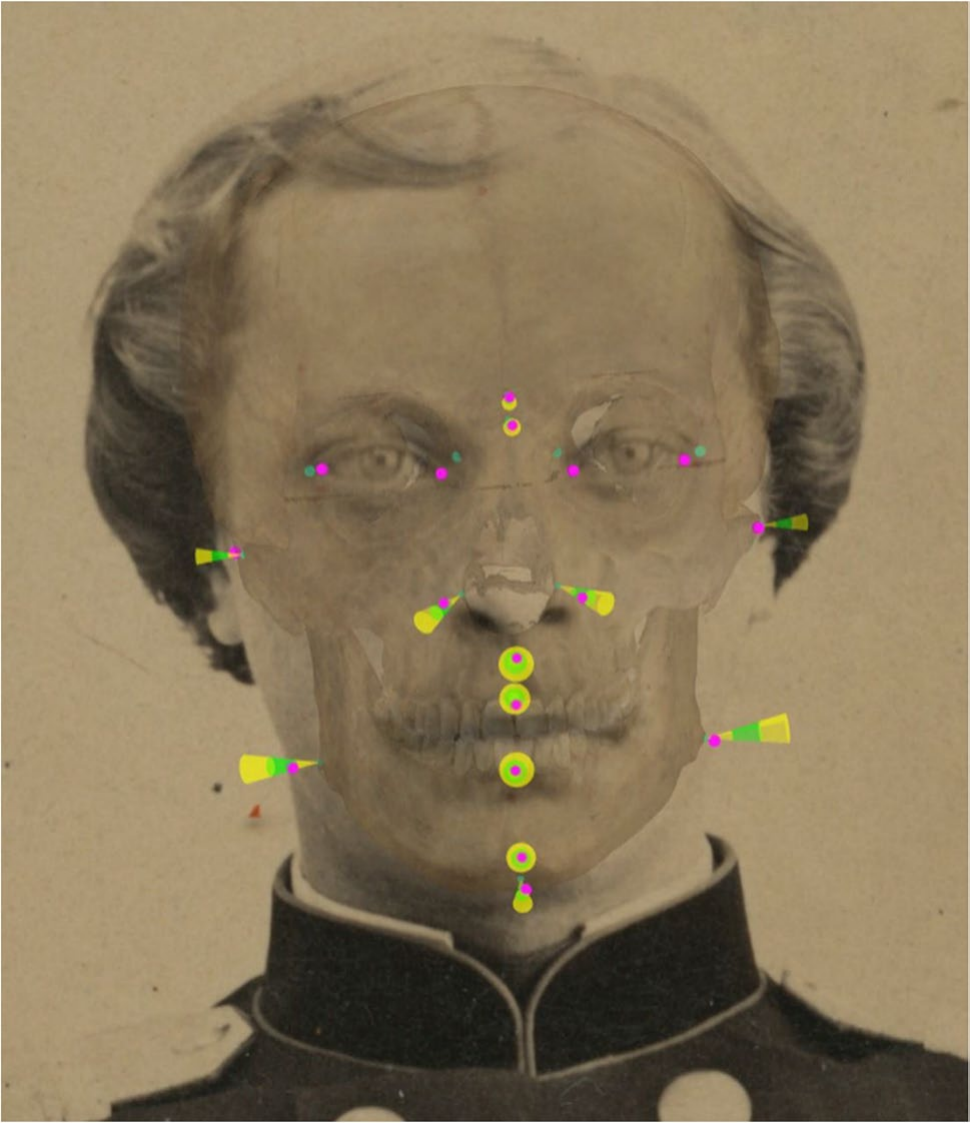
Display and evaluation of the soft tissue thickness consistency using colored 3D cones in Skeleton-ID. For each cephalometric landmark (in magenta), the area that would correspond to its craniometric pair is extracted from the soft tissue thickness study that we have selected, and then represented by a 3D cone. The green area covers all values within the mean ± the standard deviation (n’ – n, al’ L – al L, al’ R – al R, sn’ – ss, ls’ – pr, li’ – id, pg’ – pg, gn’ – gn). The yellow area covers values that are 3 times the standard deviation below or above the mean (zy’ L – zy L, zy’ R – zy R, go’ L – go L, go’ R – go R). The landmark pairs en’ – d and ex’ – ec were not considered due to insufficient data for statistical testing. Note that the size of the soft tissue markers (cones) in the images is affected by the same camera parameters projecting the skull 3D model

- Opacity tool, to change the skull model’s transparency, and wipe tool, to gradually hide parts of the skull, in order to assess the morphological and positional correspondence of anatomical structures (Fig. 3).
- Display and analysis of the soft tissue depth consistency using 3D cones (Fig. 4). Their size will be determined by the soft tissue depth values from the study employed, and they will be divided in two different colored regions: yellow for those that are up to three times the standard deviation over or below the average value. The yellow region should cover 99.7% of the distribution, according to Chebyshev’s theorem, which assumes a normal distribution of the data. Green for those values that are within the average ± the standard deviation. To evaluate the consistency of each landmark, the conjunction of the cephalometric landmark with each of the cone’s regions will determine the consistency/inconsistency of the pair.

Finally, after the appropriate morphological assessments, a degree of support for the decision was assigned (limited, moderate, or strong) for each particular CFS case, according to the quantity and quality of the available materials (AM photographs, mandible, and skull) following the criteria laid out by MEPROCS [26].

## Results

**Fig. 5:**
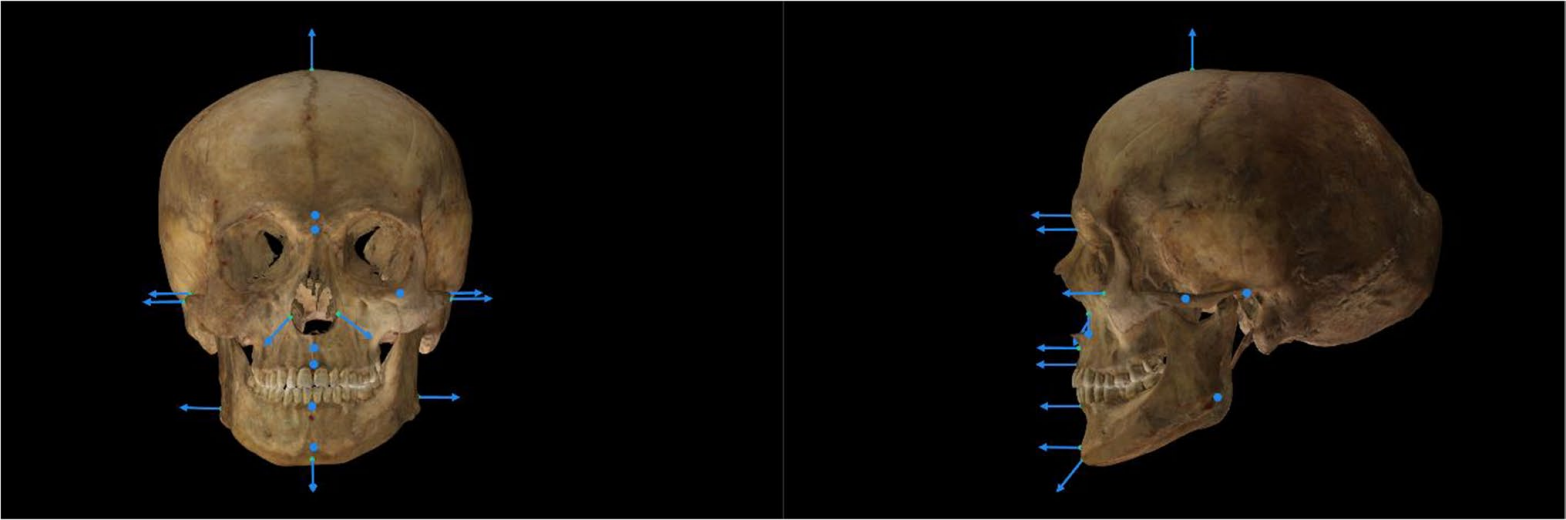
Representation of the soft tissue direction through a directional vector for each craniometric landmark in a skull 3D model in frontal and lateral view

As explained before, all participants made use of Skeleton-ID, which includes the POSEST-SFO algorithm [15]. On the one hand, the solution provided by this algorithm relies on the soft tissue depth study employed, Carl Stephan’s meta-study [22] was used in all the cases and by all the participants. On the other hand, POSEST-SFO is guided by the location of cephalometric landmarks, a set of (x,y) coordinates in a facial photograph, and the location of the corresponding craniometric landmarks, a set of (x,y,z) coordinates in a 3D skull model. Both sets of landmarks were manually located by each individual expert. Additionally, it also requires a soft tissue direction, i.e., a directional vector with origin in each craniometric landmark, pointing to the theoretical spatial location of the corresponding cephalometric landmark (Fig. 5). The direction of this vector is initially calculated by Skeleton-ID as being perpendicular to the surface of the 3D bone model. However, a manual refinement of this direction is required in many cases due to a noisy acquisition or because of the drastic morphological changes of the surface of the skull in certain regions (orbits, piriform aperture, lower part of the mandible, etc.). Thus, before analyzing the identification results (“Decision-making” section), we provide an analysis of the variability in location of cephalometric and craniometric landmarks (“Localization of cephalometric and craniometric landmarks” section) as well as establishing the direction of the soft tissue (“Establishing soft tissue vector direction” section).

### Localization of cephalometric and craniometric landmarks

This section focuses on the variability in the location of cephalometric landmarks on the photographs and craniometric landmarks on the skull 3D models.

Distances measured over digital photographs are relative to their resolution and to the relative size of the face in the image. Therefore, to average results from different images, each distance must be normalized (scaled) with respect to another measurement over the same image, such as the width or height of the face. In this study, the variability scores of each photo have been normalized with respect to the distance between left and right exocanthion (a proxy for the width of the face); they are thus reported as percentages of the latter distance. Finally, values corresponding to bilateral landmarks have been aggregated.

In order to study a potential impact on the inter-observer location error due to the use of the ad hoc landmark location tools provided by Skeleton-ID, Table 5 shows a comparison between the mean variability scores obtained in this study when considering all participants and the results obtained by Valsecchi [25]. We have also reported the *p* values obtained by performing an independent *t*-test for each landmark followed by adjustment for multiple comparisons using Holm’s method. The variability scores obtained in the current study are lower than those of [21] for 11 out of 13 landmarks. Very large differences (8–11%) are observed when considering zygion, gonion, and vertex, while that of gnathion is just above 5%. As for statistical significance, there are six occurrences of *p* values being lower than 0.05, with five occurrences corresponding to lower variability scores obtained in the current study.

Similarly, the variability in the location of craniometric landmarks on 3D skull models was analyzed. The variability scores are reported in millimeters and values corresponding to bilateral landmarks have been aggregated in Table 4. The greatest variability is observed in five of the 13 landmarks with a dispersion of more than 2 mm (ectoconchion, gnathion, gonion, zygion, and vertex).

**Table 4:** Normalized inter-observer variability of the localization of cephalometric landmarks using Skeleton-ID in the current study (second column) and in [23] (third column). The fourth column reports the adjusted *p* value of a series of *t*-tests. The fifth column represents the mean inter-observer variability in the localization of craniometrics landmarks using Skeleton-ID in 3D skull models, measured in millimeters. Each row corresponds to a different landmark

Cephalometric landmark	Using Skeleton-ID	Campomanes-Álvarez et al., 2015	*p* value	Craniometric landmark	Using Skeleton-ID
Alare	2.74	2.873	0.42	Alare	1.293
Endocanthion	3.023	2.668	0.007	Dacryon	1.64
Exocanthion	2.845	2.719	0.42	Ectoconchion	2.137
Glabella	4.098	4.414	0.31	Glabella	1.856
Gnathion	5.944	11.19	0.64	Gnathion	2.087
Gonion	7.266	15.491	0	Gonion	2.369
Labiale inferius	1.917	3.628	0.001	Infradentale	1.386
Labiale superius	2.575	3.079	0.08	Prosthion	0.962
Nasion	3.316	5.611	0.11	Nasion	0.758
Pogonion	4.515	8.205	0.02	Pogonion	1.551
Subnasale	2.368	2.525	0.33	Subspinale	1.83
Vertex	10.329	21.514	0	Vertex	7.402

### Establishing soft tissue vector direction

In addition to studying the dispersion in the location of cephalometric and craniometric landmarks, we carried out a study of the variation in the orientation of vectors, which as previously explained, illustrates the direction of the soft tissue in-between each pair of landmarks.

It is relevant to note that the information regarding the direction of soft tissue in-between pairs of facial and cranial landmarks is derived from few existing graphical representations displaying the location of these landmarks and the trajectories used when measuring soft tissue thickness [20, 27, 28]. However, most soft tissue depth studies do not include any reference to the direction of the measurement taken on each point, or to how the head is oriented in order to take the measurements, which means it is highly unlikely that the measures taken were perfectly perpendicular.

The variability scores are reported in angles (in degrees), and values corresponding to bilateral landmarks’ vectors have been aggregated. The values in Table 5 represent the variation in angles between the vectors oriented by the five participants.

**Table 5:** Mean inter-observer variability in the orientation of vectors on 3D skull models, measured in degrees

Craniometric landmark (origin of the vector)	Angle between vectors
Alare	23.9
Dacryon	22
Ectoconchion	28.1
Glabella	8.4
Gnathion	15.2
Gonion	32.6
Infradentale	16.4
Menton	10.2
Nasion	13.6
Pogonion	10.9
Prosthion	12.2
Subspinale	15.2
Vertex	5.6
Zygion	7.8

### Decision-making

Considering the examined materials and the consistency of the correspondences between skulls and faces, a final decision was provided for each CFS case. Each of the five participants provided a degree of support within the gradual support scale established by MEPROCS consortium. This scale assigns a value in terms of strong, moderate, or limited support to the assertion that the skull and the facial image belonged (or not) to the same person. Other discriminatory features, such as asymmetries, were also considered for the modification of the final degree of support.

To assess the performance of the decision-making, we rely on the identification results which have been obtained through the application of different identification techniques and the analysis of circumstantial evidence reported in Table 6. Of the total of 198 CFS cases, 9 cases were positive and 189 negatives. Next, for each participant, we consider the number of decisions made with respect to the total number of cases with strong, moderate, and limited support and calculate the following four quantities:

- the ratio of cases where a positive case has been classified correctly as positive (true positive)
- the ratio of cases where a positive case has been classified incorrectly as negative (false negative)
- the ratio of cases where a negative case has been classified incorrectly as positive (false positive)
- the ratio of cases where a negative case has been classified correctly as negative (true negative)

**Table 6:** Identification results obtained by other techniques. The third column contains the different identification methods that were used or taken into consideration while establishing the positive identification

AM case	PM case	Identification method
AM1	–	• Remains were not recovered from the burial pits (DNA verified during testing with samples of two living descendants)
AM3	K4P7	• Archeological context: a pit that contained the remains of two individuals who had been executed on the same day and buried together; the other individual had been excluded through genetic identification—DNA testing with the sample from niece; relations with other individuals excluded • Age (AM—23 years, PM—23–26 years), sex, and execution method (fusillade) concur • Other identities excluded by craniofacial superimposition
AM4	K12P18	• Archeological context: single burial (single execution) • Age (AM—26 years, PM—25–34 years), sex, and execution method (not fusillade—i.e., hanging) concur • DNA testing (relations with other individuals excluded) • Other identities excluded by craniofacial superimposition
AM6	K3P5	• Archeological context: single burial (single execution); wedding ring with initials and ceremony date • Age (AM—37 years, PM—30–39 years), sex, pathologies (AM—wounded lumbosacral area 13 months before death, PM—traces of healing trauma on lumbar vertebrae and sacrum), and execution method (not fusillade—i.e., hanging) concur • DNA testing (relations with other individuals excluded) • Other identities excluded by craniofacial superimposition
AM7	K13P19	• Archeological context: single burial (single execution) • Age (AM—31 years, PM—25–34 years), sex, and execution method (fusillade) concur • DNA testing (relations with other individuals excluded) • Other identities excluded by craniofacial superimposition
AM12	K2P4	• Archeological context: triple burial (the only instance in which 3 people had been executed on the same day) • Age (AM—26 years, PM—25–30), sex, and execution method (not fusillade—i.e., hanging) concur • DNA testing (relations with other individuals excluded) • Other identities excluded by craniofacial superimposition • Identities of two others excluded by craniofacial superimposition
AM17	K6P11	• Archeological context: 2 individuals executed on the same day, buried with the remains of AM 18 (double burial) • Age (AM—22 years, PM—20–26 years), sex, and execution (not fusillade—i.e., hanging) method concur • DNA testing (relations with other individuals excluded) • Other identities excluded by craniofacial superimposition
AM18	K6P10	• Archeological context: 2 individuals buried on the same day, found alongside AM17 (double burial) • Age (AM—27 years, PM—25–34 years), sex, and execution method (not fusillade—i.e., hanging) concur • DNA testing (relations with other individuals excluded) • Other identities excluded by craniofacial superimposition
AM19	K14P20	• Archeological context (single burial—single execution) • Age (AM—28–29 years, PM—30–39 years), sex, and execution method (fusillade) concur • DNA testing (relations with other individuals excluded) • Other identities excluded by craniofacial superimposition
AM20	K8P13	• Archeological context: single burial (single execution) • Age (AM—26 years, PM—20–25 years), sex, and execution method (not fusillade—i.e., hanging) concur • DNA testing (relations with other individuals excluded) • Other identities excluded by craniofacial superimposition
AM21	K1P1	• Remains were not recovered from the burial pits (DNA verified during testing with samples of two living descendants)

The results of this analysis are reported in Table 7 for the different degrees of support.

**Table 7:** Performance of the five participants on decisions made with strong, moderate, and limited support. Number of positive cases out of 9 in the total number of decisions made (# Positive), true positives ratio (TP), false positives ratio (FP), number of negative cases out of 189 in the total number of decisions made (# Negative), true negatives ratio (TN), and false negatives ratio (FN)

Participant	# Positive	TP	FP	# Negative	TN	FN
**Decisions made with strong support**						
P1	–	–	–	–	–	–
P2	0	0%	100%	1	0%	100%
P3	1	100%	0%	29	100%	0%
P4	1	100%	0%	17	100%	0%
P5	1	100%	0%	17	100%	0%
**Decisions made with moderate support**						
P1	2	50%	1.96%	51	98.03%	50%
P2	1	0%	10.34%	29	89.65%	100%
P3	3	100%	0%	53	100%	0%
P4	2	100%	0%	52	100%	0%
P5	3	100%	0%	55	100%	0%
**Decisions made with limited support**						
P1	6	0%	4.44%	135	95.55%	100%
P2	0	0%	13.51%	37	86.48%	0%
P3	0	0%	0%	4	100%	0%
P4	4	75%	2.45%	122	97.54%	25%
P5	3	100%	2.40%	83	97.59%	0%

## Conclusions and discussion

This is the first multi-center CFS blind validation study on a real-life, complex identification scenario with multiple comparisons, pertaining to a mass grave that contained 18 human skulls. In addition, this study has proposed the validation of the first tool specifically designed for CFS, Skeleton-ID™—meeting the MEPROCS requirements and standards for CFS software—, together with the automatic POSEST-SFO algorithm [15] the software includes.

In the case of CFS, the correct location of the craniometric and cephalometric landmarks is essential to guide the skull-face overlapping process or even to assess the consistency of soft tissue thickness once both images have been overlapped. However, very few studies [21, 29] have addressed the problem of accurately locating cephalometric landmarks in photographs when these play an important role in the forensic identification process. In this study, we have carried out an inter-observer variability analysis for 13 cephalometric landmarks in 14 AM photographs. Additionally, we have also analyzed the inter-observer variability of the craniometric landmarks for the 18 3D skull models.

Regarding the variability of the cephalometric landmarks, the results of this analysis indicate that gnathion, gonion, zygion, and vertex landmarks present a greater dispersion, which agrees with the results obtained by [21] in their study. In addition, the results obtained in that study have been compared with those obtained by the five participants, revealing significant differences in terms of the variability of gonion, labiale inferius, pogonion, vertex, and zygion landmarks, which present less dispersion in this case. This may be due to the use of the specific ad hoc landmark location tools provided by Skeleton-ID (crosshairs and auxiliary lines) that allow a more precise refinement in the placement of the cephalometric landmarks in photographs. For example, using the vertical auxiliary line to determine the midsagittal plane of the face when placing the medial landmarks.

In the case of the craniometric landmarks, the ectoconchion, gnathion, gonion, zygion, and vertex landmarks present the greatest dispersion with values greater than 2 mm. In the case of 3D models, Skeleton-ID tools are even more precise when placing craniometric landmarks. Using the auxiliary lines, it is possible to accurately determine the most anterior or posterior point of an anatomical structure based on the definition of each landmark once the Frankfurt plane has been established. On the other hand, the 4-screen view allows to refine the position of the landmark in all views of the skull (lateral, frontal, basal, and occipital).

In regard to the orientation of the vectors, the variability was measured as the difference in angle (in degrees) between the vectors oriented by the different participants. The greatest dispersion was found in bilateral landmarks, particularly in the landmarks around the orbital area (ectoconchion and dacryon), for which there is conflicting information regarding the relationships of the bony and soft tissues. The direction of the cephalometric homolog for each craniometric landmark in this area would be influenced both by eyeball protrusion, not easily assessed in skull 3D models, and with limited studies carried out in the literature [31] and points of attachment of the palpebral fissure ligaments [32, 33] as well as possible variation associated to aging [34].

Considerable dispersion was also found in the gonion landmark vector orientation, which might be related both to the variability in the placement of the craniometric landmark among the participants and to inconsistencies in the methodology followed to orient these vectors. This was probably further emphasized by the presence of notable gonial eversion in a great number of skulls of the sample. Similarly, a considerably high dispersion was found in the orientation of the vector of alare. Estimating the direction of the soft tissue at these points is a complex task because of the position of the nasal alae in relation to them, slightly anterior and inferior (approximately 6 mm anteriorly and 4 mm inferiorly according to [3]).

Although there was considerably less dispersion found among the medial landmarks, it was still noteworthy. One possible cause behind this could be the existing variation in the localization of the orbitale and porion landmarks, which are used to establish the Frankfurt plane. Thus, any variation in the rotation of the skull would subsequently cause a variation in the angle of the vectors as they are oriented perpendicular to the craniometric landmark. The accurate establishment of the Frankfurt plane seems to be a complex task on skulls, and it may prove far more uncertain when dealing with live subjects, where the lower rim of the orbit is not visible. However, this problem has not been addressed in the literature (apart from [35]) and is not even mentioned in most facial soft-tissue depth studies, with authors assuming an approximation of the Frankfurt plane and providing measurements that are not considering neither the plane used to take them or the uncertainty in measurement.

As the POSEST-SFO algorithm [9] uses both the position of the cephalometric and craniometric landmarks and the direction and length of the vectors, the resulting overlay for each comparison were slightly different for each of the participants. Although the overlay can be manually refined, this requires some knowledge of craniofacial anatomy as well as of camera parameters in order to obtain good results. In future research, it may prove of interest to study the differences between the overlays obtained by different researchers and analyze how this may be influencing the decision-making stage and the subsequent identifications.

Regarding the correspondence decisions made by the five participants for the comparisons made, these have been given in terms of limited, moderate, or strong support according to the MEPROCS requirements that must be met for each degree.

Considering the decisions made with a strong degree of support, the highest within the MEPROCS scale, the three anthropologists (P3, P4, and P5) reached correct conclusions in 100% of the cases, in a total of 30, 18, and 18 decisions made, respectively. On the other hand, regarding the students, P1 did not make any decision in this degree of support and P2 only classified one case as positive, turning out to be a false positive. It is important to note that, in order to make decisions with strong support, the skull must be preserved complete and there must be at least two photographs of the subject of sufficient quality and in different views. This was the case of subject AM6, for whom four photographs of sufficient quality were available in different views and in which a remarkable asymmetry could be observed in the left orbital area that was also present in skull K3P5. Considering this information, this was the only positive case classified with strong support by the anthropologists.

Regarding the degree of moderate support, one of the requirements to be able to make this decision is that there is at least one photograph of sufficient quality, this being the case for seven of the 11 subjects in the sample. The five participants made between 30 and 58 decisions within this degree of support, again obtaining 100% of correct decisions in the case of P3, P4, and P5. On the other hand, P1 and P2 made errors of 1.96% and 10.34% of FP and 50% and 100% of FN on the decisions made.

Finally, the limited support degree is assigned when only one photograph is available, and it is of poor quality. In this case, the five participants have made between four and 141 decisions. In this degree of support, the three anthropologists who had 100% of correct decisions with a strong and moderate degree of support made errors between 0 and 2.45% of FP and between 0 and 25% of FN. It is important to note that the low error rate of P3 (0% of FP and FN) is due to the number of decisions made (4) with respect to P4 (126) and P5 (86). In the case of students, P1 made errors of 4.44% FP and 100% FN, while P2 got 13.51% FP and 0% FN.

The main difference between the identification results obtained in decision-making between anthropologists and students seems to lie in the knowledge and previous experience in CFS. The three anthropologists had specific background in Physical and Forensic Anthropology, with knowledge of craniofacial anatomy and previous experience in solving cases. In contrast, although both students received training materials on the CFS technique, neither had previous Forensic Anthropology academic background or experience with the technique. This may have led to worse results.

It should be noted that the identification process was approached in the first instance as a closed scenario by P5 (the first participant to address the cases and complete the study), where the 11 individuals in the photographs had to be among the 18 analyzed skulls. However, investigations conducted in parallel with this study revealed that this was not the case. The skulls corresponding to subjects AM1, AM12, and AM19 were not among the 18 3D models provided to the participants. Thus, the two cases classified as false positives by P5 were decided based on the few criteria that could be positively correlated, considering the low quality of the photographs and their consistency among the 18 skulls available. Subsequently, the rest of the participants were previously informed to address the identification problems as an open scenario where the subjects of the photographs could not be found among the 18 skulls provided. It is also important to highlight the fact that a positive decision in terms of limited support within the MEPROCS scale should not be taken as evidence of identification, but rather as complementary support to guide future research.

It is worth mentioning the case of subject AM4, whose provided photograph portrayed his brother. Even though he was not the individual depicted in the photograph, P3, P4, and P5 reached the same conclusion and assigned the same skull (K12P18) to it with a limited to moderate degree of support. One of the possible reasons behind this could be that since they were brothers, their facial morphology could have been very similar and considering the low quality of the AM image used for comparison, the analyzed general morphological criteria could have shared similarities. Although some studies have attested the likeness of craniofacial morphology in twins [30, 31], based on our own knowledge, no research has been conducted concerning skull-face comparisons among siblings, offspring, or close family members.

It is important to remark that the results obtained through the application of CFS in this study have been compared with the information from the chain of evidence (historical records, method of execution, age, personal elements, etc.) that have served to get to an identification. For this reason, one of the main limitations of the study has been the impossibility of validating the results obtained with other primary identification methods (i.e., DNA). Furthermore, these results suggest that both the quality of the materials used and the previous experience of the analyst play a fundamental role when reaching conclusions using the CFS technique. This is a complex technique that requires extensive knowledge of craniofacial anatomy, understanding of physical aspects of photograph capture, and previous experience in solving cases with known identity. In this study, many drawbacks had to be overcome related to the materials used, increasing the difficulty of the scenario even further and, quite certainly, negatively impacting the decision-making stage.

- No access to the original skulls was granted (only to the 3D models).
- Teeth were not visible in the photographs (preventing dental comparison).
- Only one photograph in one pose was available for 10 out of the 11 subjects.
- The photographs used were old (over 150 years old), so there was no metadata associated to them (camera model and focal lens used) and the quality of some of them was not sufficient for CFS.
- The results of the identifications have not been validated with other primary methods such as DNA.

The results obtained seem to advise cautious use of the technique when the quality and quantity of the materials is not adequate (limited support), but they also suggest that it is a reliable technique in cases with an acceptable or good quantity and quality of the materials and/or in cases with unusual morphological features (as the asymmetry observed in the skull K3P5 and the AM6 subject photographs). All these limitations can be easily overcome nowadays thanks to the widespread use of cameras in smartphones. It seems logical to think that in contemporary cases, we might be able to access dozens of recent digital photographs (including camera parameters such as the focal length), in different poses, with sufficient quality and even with partially visible dentition for dental comparison.

Another truly relevant factor that should not be overlooked is the time required to conduct the study (Table 8). The use of Skeleton-ID™ has allowed for participating researchers to locate the craniometric and cephalometric landmarks, carry out 252 SFOs (18 3D skull models × 14 facial photographs), analyze the morphological correspondences, and reach conclusions on most of the studied cases. The immense potential of the tools and the automatic algorithm based on Artificial Intelligence resides in their ability to perform tasks that are tedious, time consuming, and error prone for the expert.

**Table 8:** Average time investment of the five participants on each task (min) and total (hours)

2D landmarks location (min)	3D landmarks location (min)	SFO evaluation (min)	Total (hours)
370	1200	5054	110.40

The high accuracy achieved by the anthropologists exemplifies the great profit that can be harvested from implementing the use of specific software in conjunction with Artificial Intelligence algorithms and the MEPROCS framework. The results obtained semi-automatically (anthropologist with the aid of adequate graphical tools and the automatic SFO algorithm) pose a new step in the implementation of the CFS technique as a primary forensic identification technique. Both the methodological approach [11, 12, 26] and the available technology [15, 19, 32] have greatly advanced in the last few years and show their value even in highly complex scenarios such as the one target of this study. The future of the CFS technique goes, according to the authors’ opinions, through the following steps:

- The need for multidisciplinary (facial anatomy/morphology, photography/photogrammetry) and specific (landmark location, skull-face overlay, morphological correspondence analysis) training.
- The methodological application of the CFS technique (following good practices and standards set by the MEPROCS consortium and new to come).
- The use of software tools specifically designed for CFS.
- The use of Artificial Intelligence algorithms to support the expert in the successive stages of the process.

Concerning the last point, ongoing and future work includes the design of new Artificial Intelligence algorithms to automatically locate cephalometric landmarks in photographs [33, 34] and craniometric landmarks in 3D skull models [35], a new SFO algorithm that operates with multiple photographs simultaneously (estimating soft tissue depth thanks to triangulation principles) and a decision support system [13, 36], as well as the study of the impact of each of these implementations on the precision of the results achieved in diverse identification scenarios (historical mass graves, recent cases of missing persons, etc.).

### Supplementary Information

Below is the link to the electronic supplementary material.

Supplementary file1 (PNG 2889 KB)

Supplementary file2 (PNG 2866 KB)

Supplementary file3 (PNG 2968 KB)

Supplementary file4 (PNG 2976 KB)

Supplementary file5 (PNG 2922 KB)

Supplementary file6 (PNG 2931 KB)

Supplementary file7 (PNG 3182 KB)

Supplementary file8 (PNG 2919 KB)

Supplementary file9 (PNG 2780 KB)

Supplementary file10 (PNG 2902 KB)

Supplementary file11 (PNG 2938 KB)

Supplementary file12 (PNG 2851 KB)

Supplementary file13 (PNG 3012 KB)

Supplementary file14 (PNG 2761 KB)

Supplementary file15 (PNG 2700 KB)

Supplementary file16 (PNG 2699 KB)

Supplementary file17 (PNG 2902 KB)

Supplementary file18 (PNG 2823 KB)

## Data Availability

All the data employed in this study could be shared under certaing conditions. To access primary data (facial photographs and 3D skull models) ask Dr. Rimantas Jankauskas. To access secondary data (landmarks, vectors and resulting SFOs), contact the corresponding author.
