# Supplementary figures and images for: Computer-aided craniofacial superimposition validation study: the identification of the leaders and participants of the Polish-Lithuanian January Uprising (1863–1864)
Source: Int J Legal Med. 2022 Dec 15;138(1):107–21. doi: 10.1007/s00414-022-02929-4 (PMC10772000; doi:10.1007/s00414-022-02929-4)

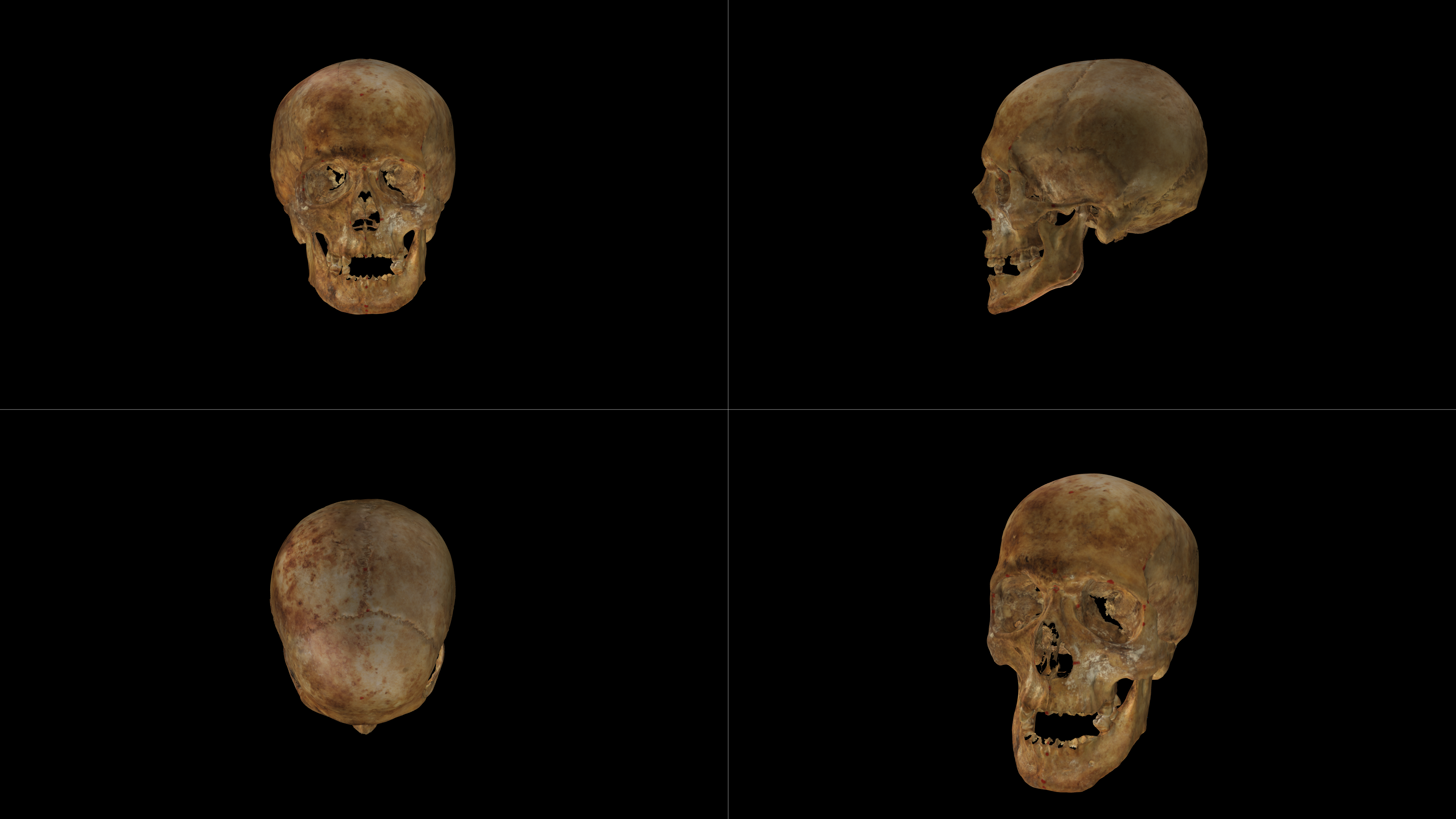

Supplement: Supplementary file 1 — Supplementary file1 (PNG 2889 KB) [file 414_2022_2929_MOESM1_ESM.png]

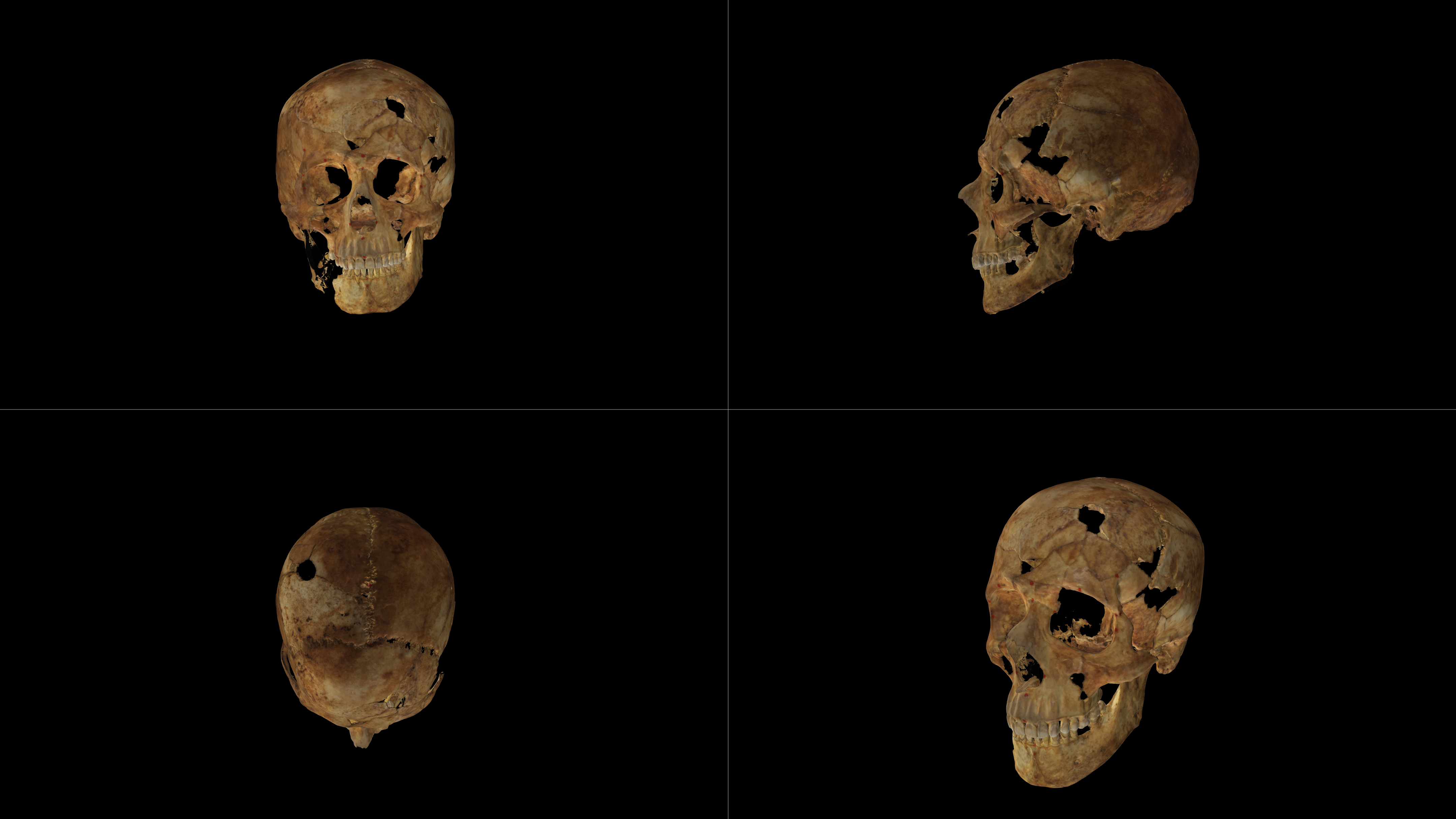

Supplement: Supplementary file 2 — Supplementary file2 (PNG 2866 KB) [file 414_2022_2929_MOESM2_ESM.png]

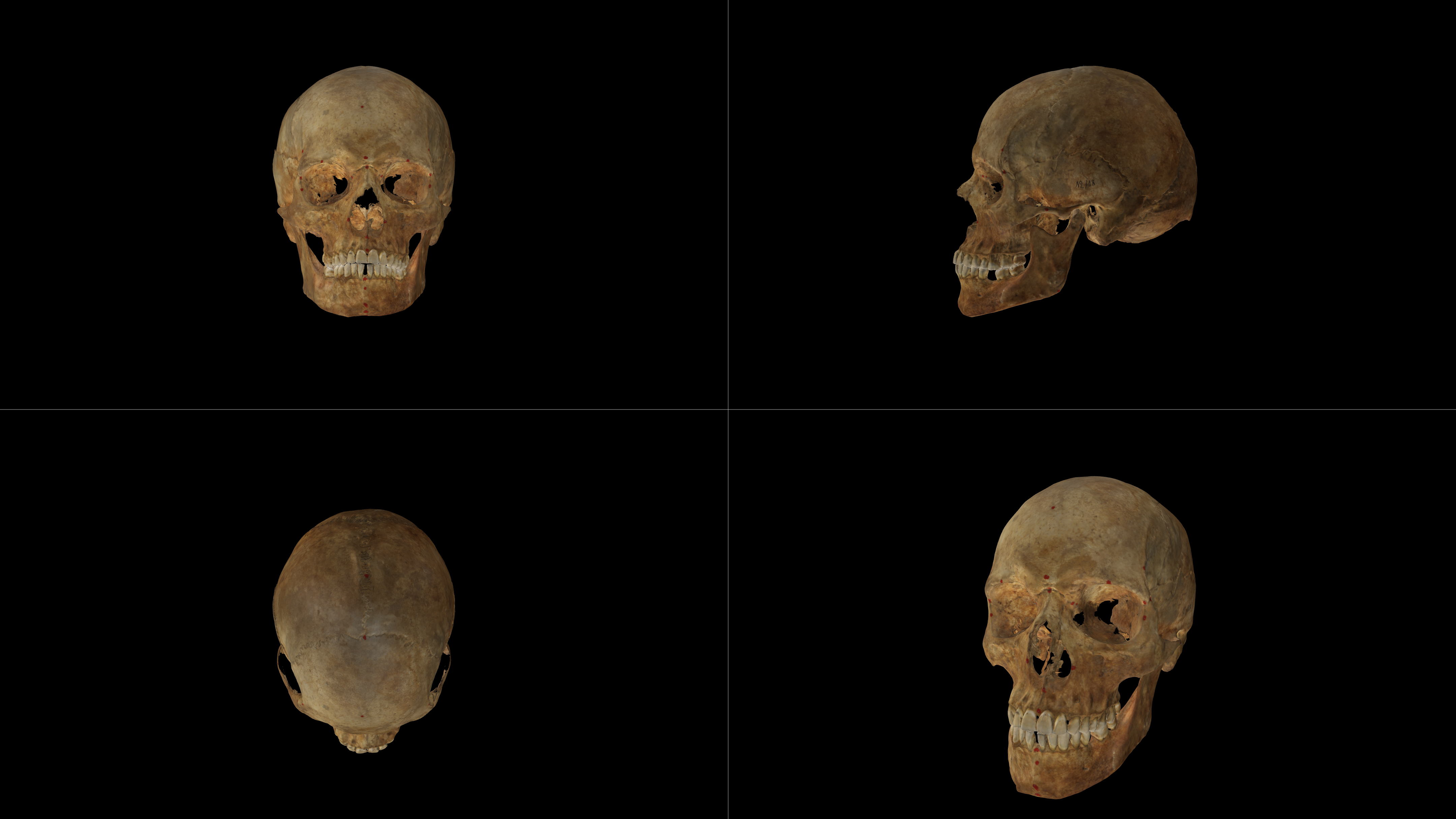

Supplement: Supplementary file 3 — Supplementary file3 (PNG 2968 KB) [file 414_2022_2929_MOESM3_ESM.png]

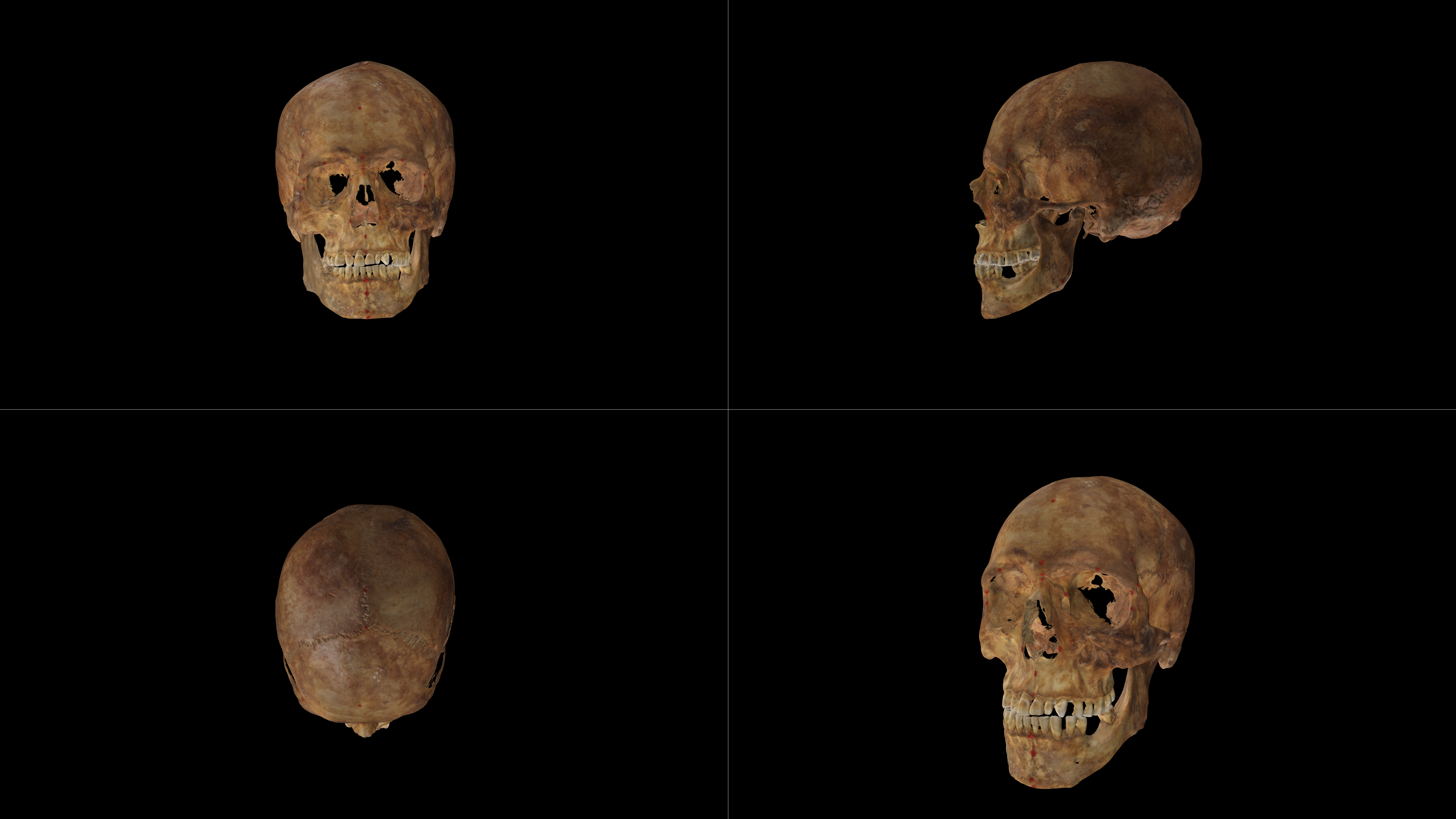

Supplement: Supplementary file 4 — Supplementary file4 (PNG 2976 KB) [file 414_2022_2929_MOESM4_ESM.png]

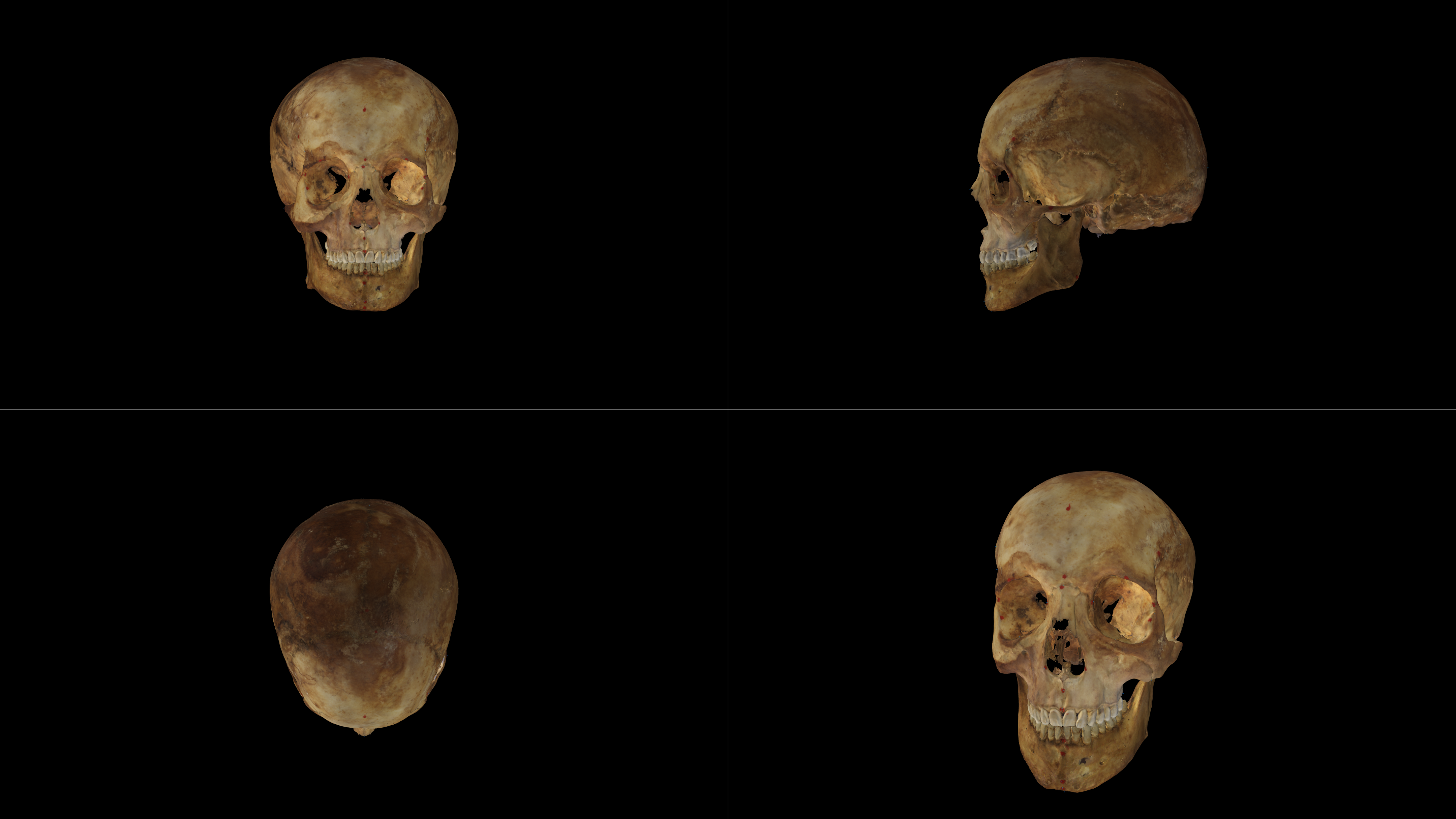

Supplement: Supplementary file 5 — Supplementary file5 (PNG 2922 KB) [file 414_2022_2929_MOESM5_ESM.png]

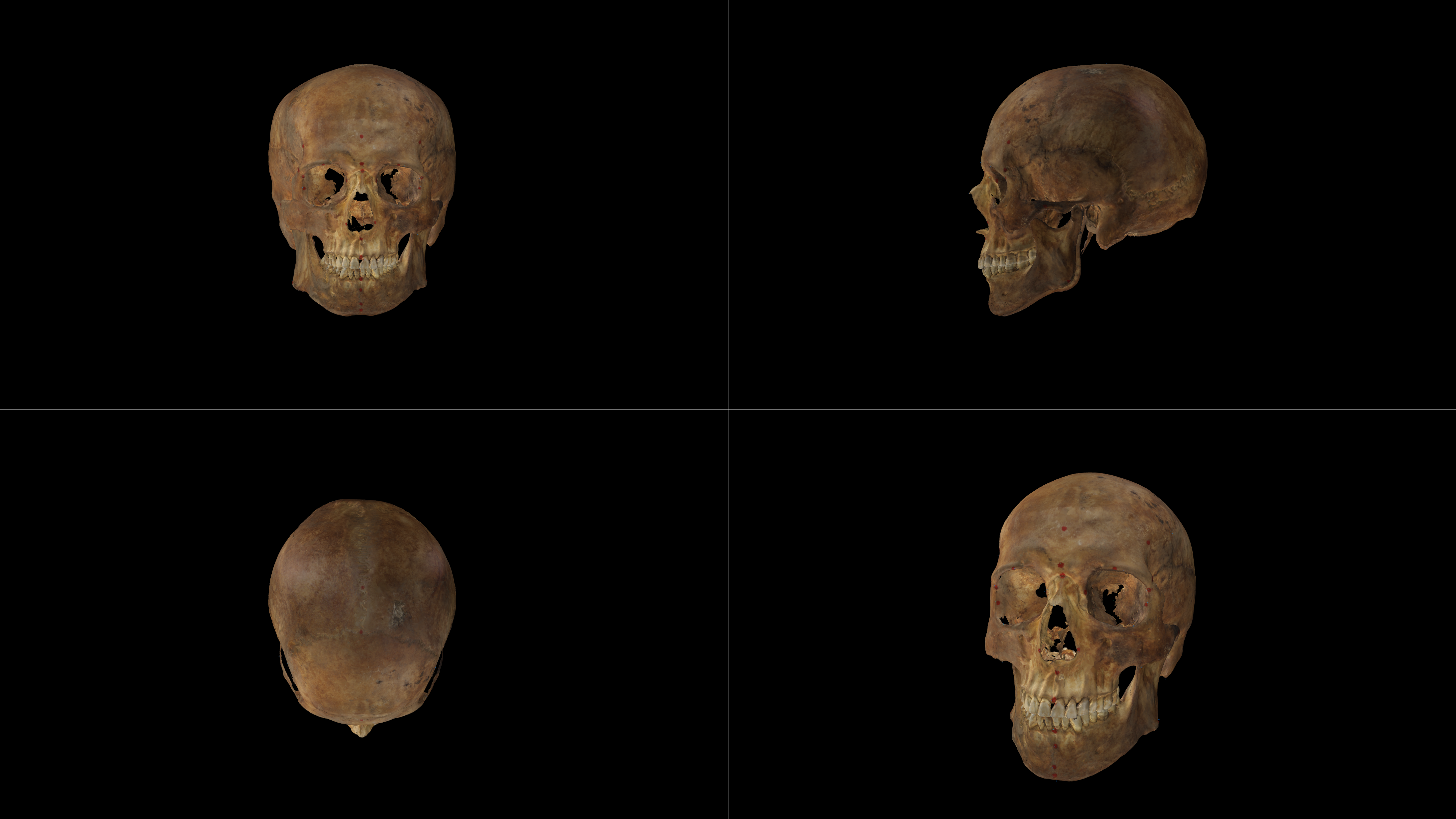

Supplement: Supplementary file 6 — Supplementary file6 (PNG 2931 KB) [file 414_2022_2929_MOESM6_ESM.png]

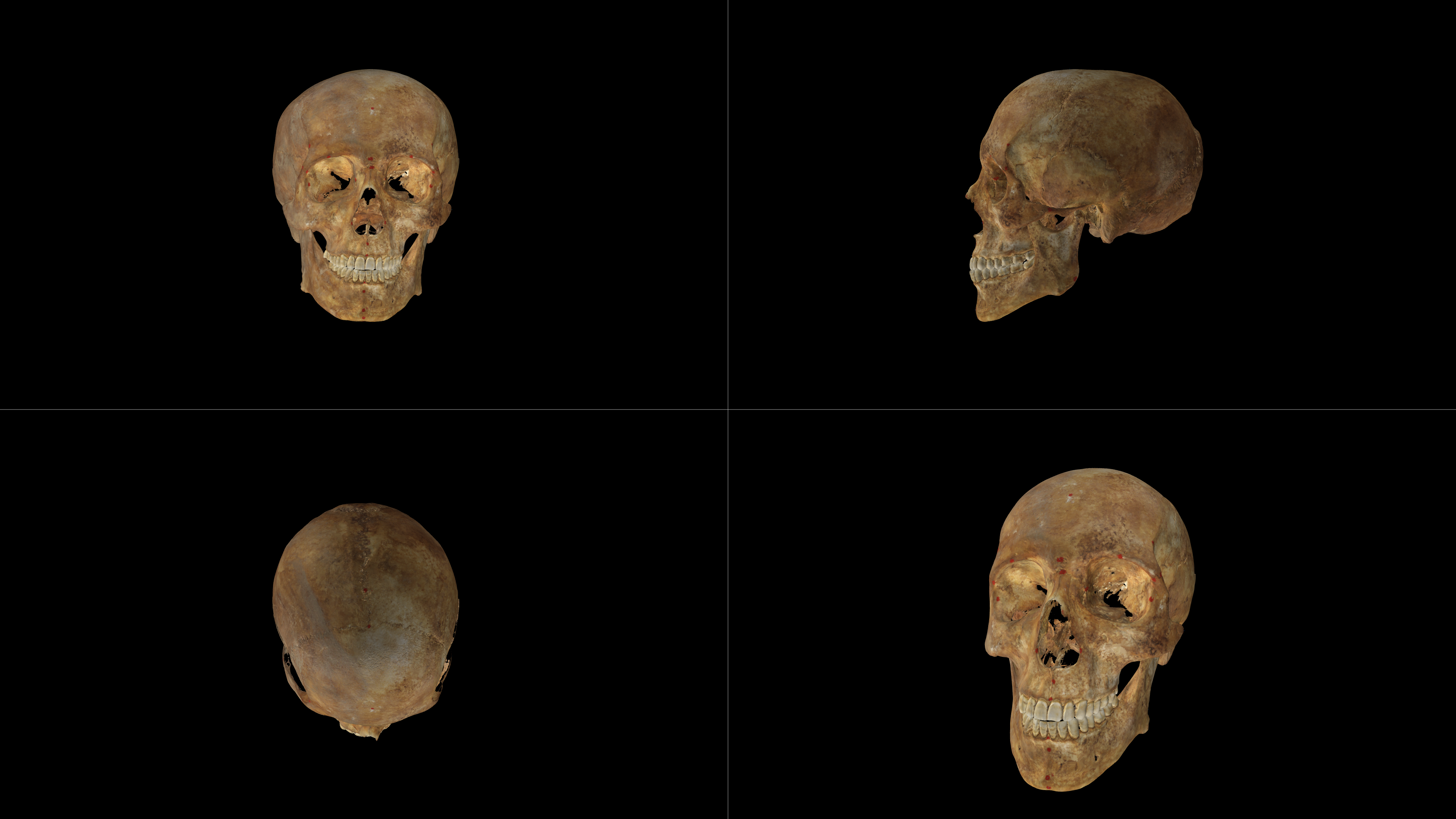

Supplement: Supplementary file 7 — Supplementary file7 (PNG 3182 KB) [file 414_2022_2929_MOESM7_ESM.png]

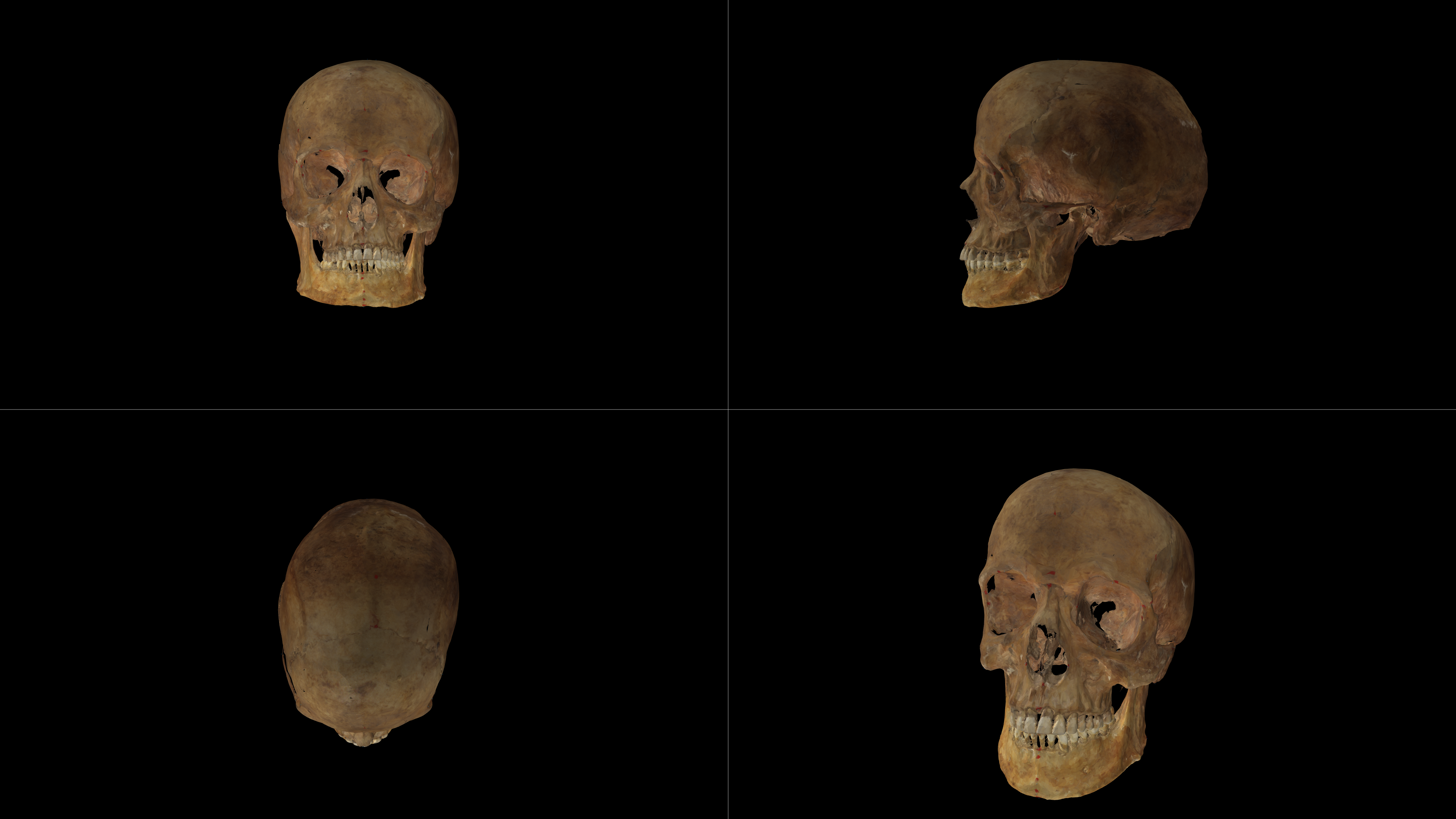

Supplement: Supplementary file 8 — Supplementary file8 (PNG 2919 KB) [file 414_2022_2929_MOESM8_ESM.png]

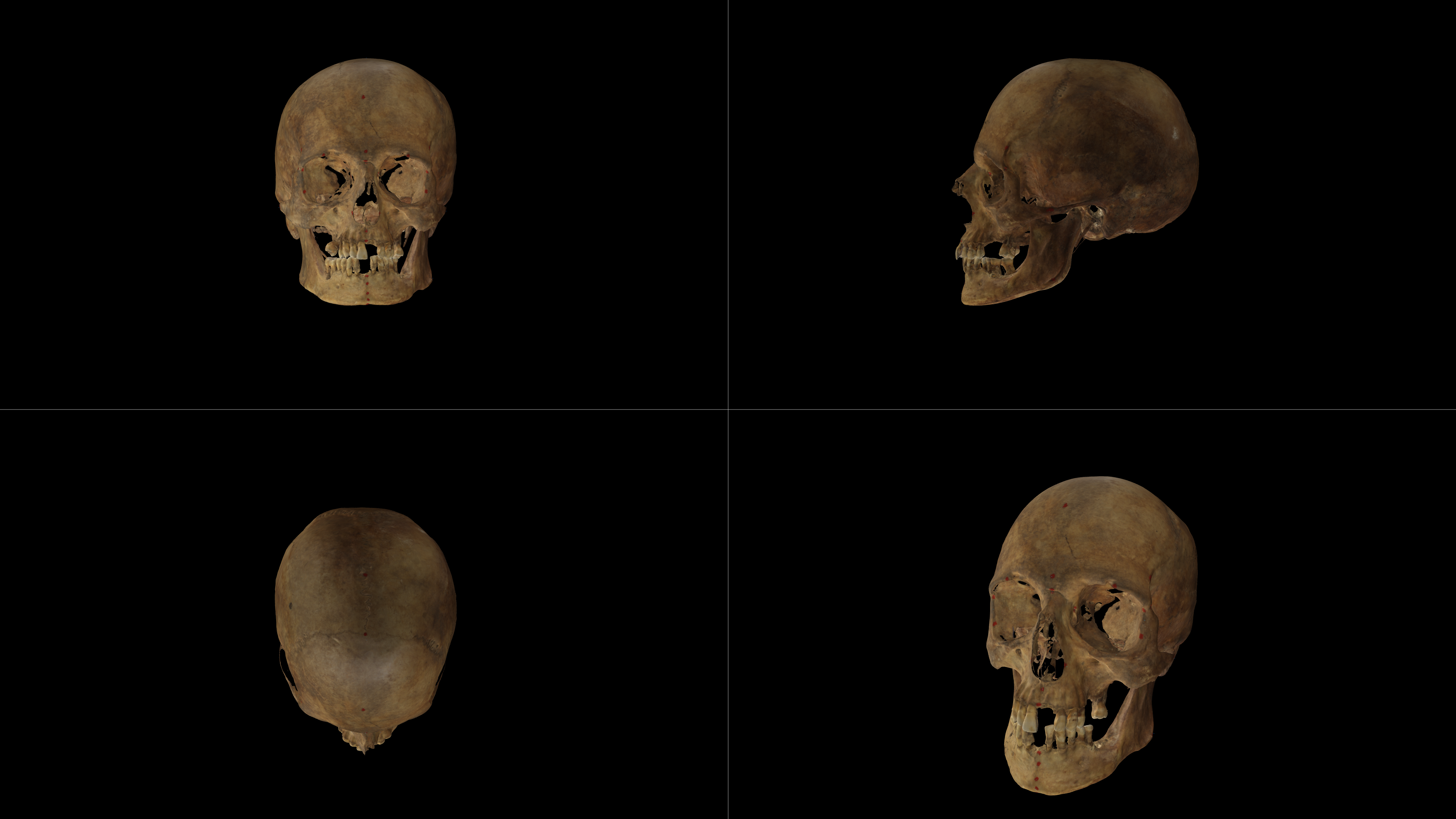

Supplement: Supplementary file 9 — Supplementary file9 (PNG 2780 KB) [file 414_2022_2929_MOESM9_ESM.png]

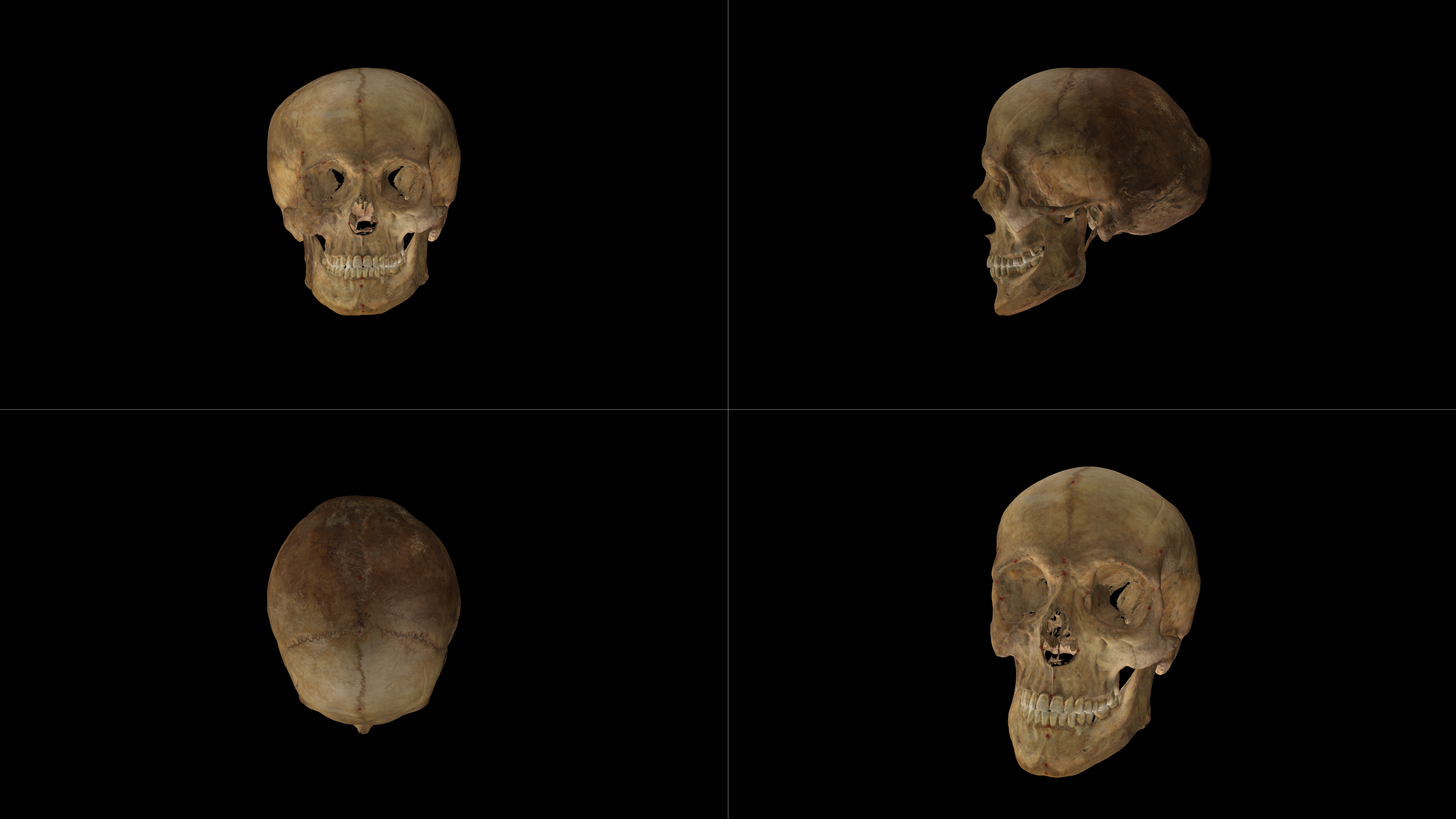

Supplement: Supplementary file 10 — Supplementary file10 (PNG 2902 KB) [file 414_2022_2929_MOESM10_ESM.png]

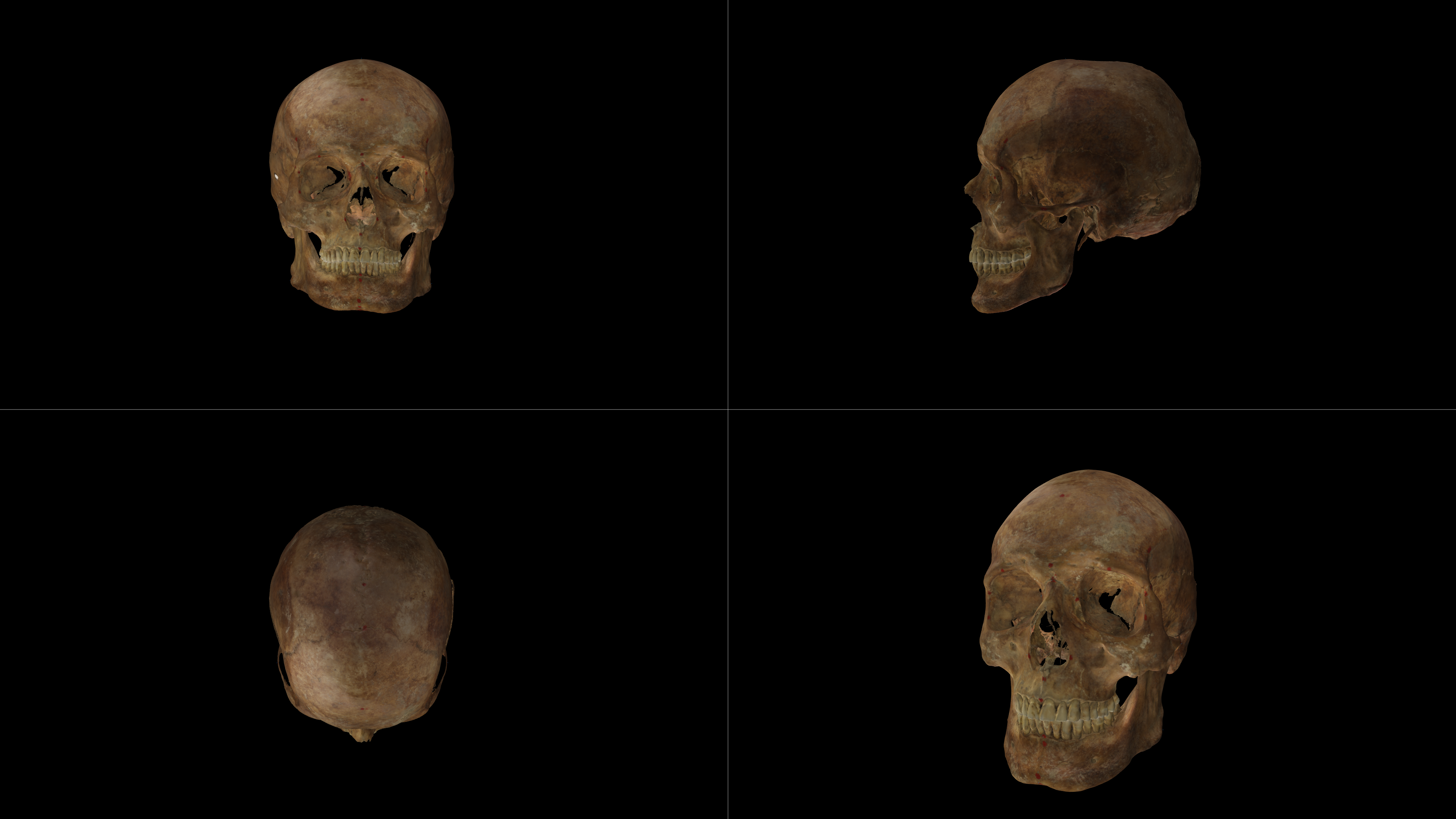

Supplement: Supplementary file 11 — Supplementary file11 (PNG 2938 KB) [file 414_2022_2929_MOESM11_ESM.png]

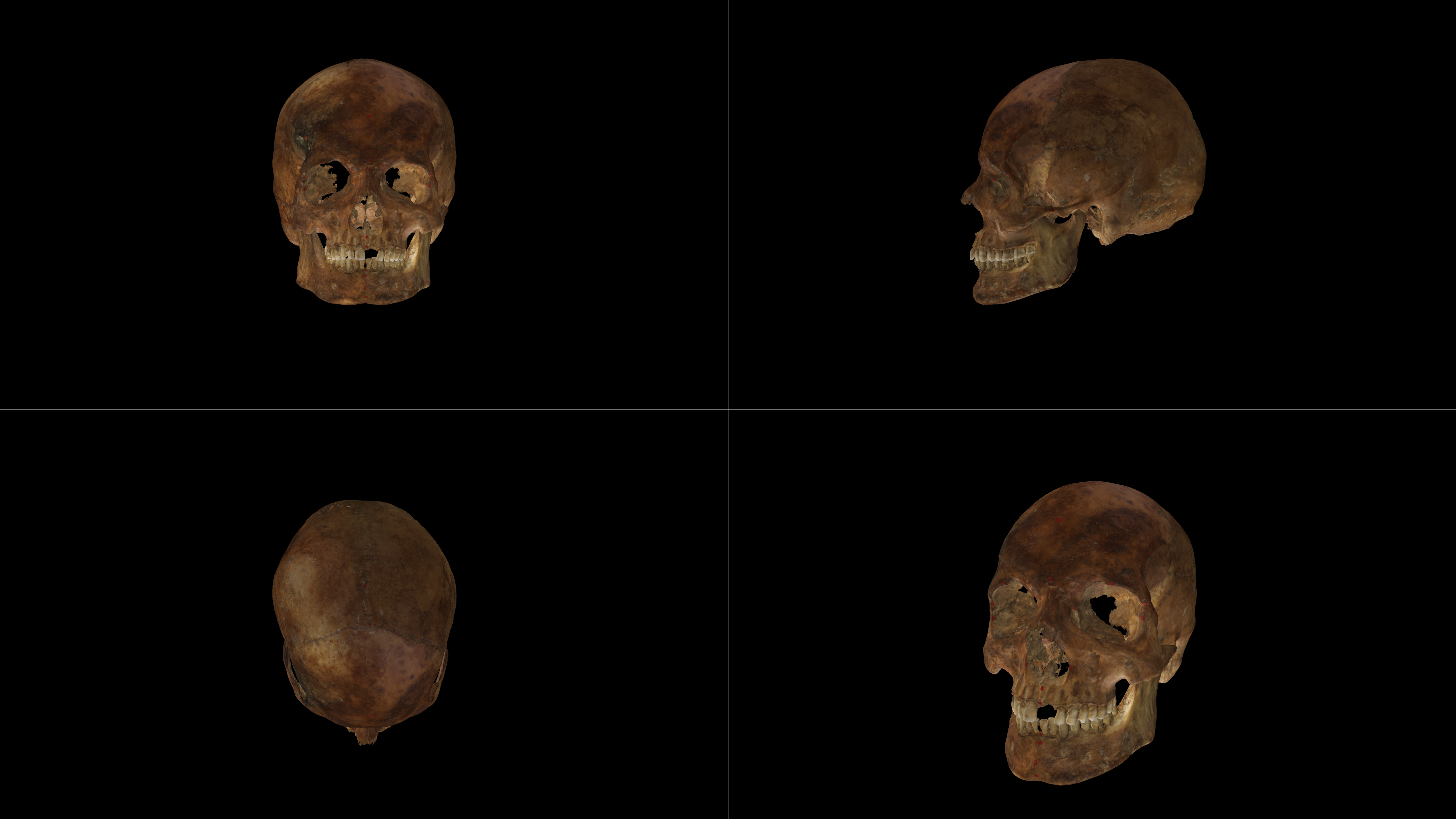

Supplement: Supplementary file 12 — Supplementary file12 (PNG 2851 KB) [file 414_2022_2929_MOESM12_ESM.png]

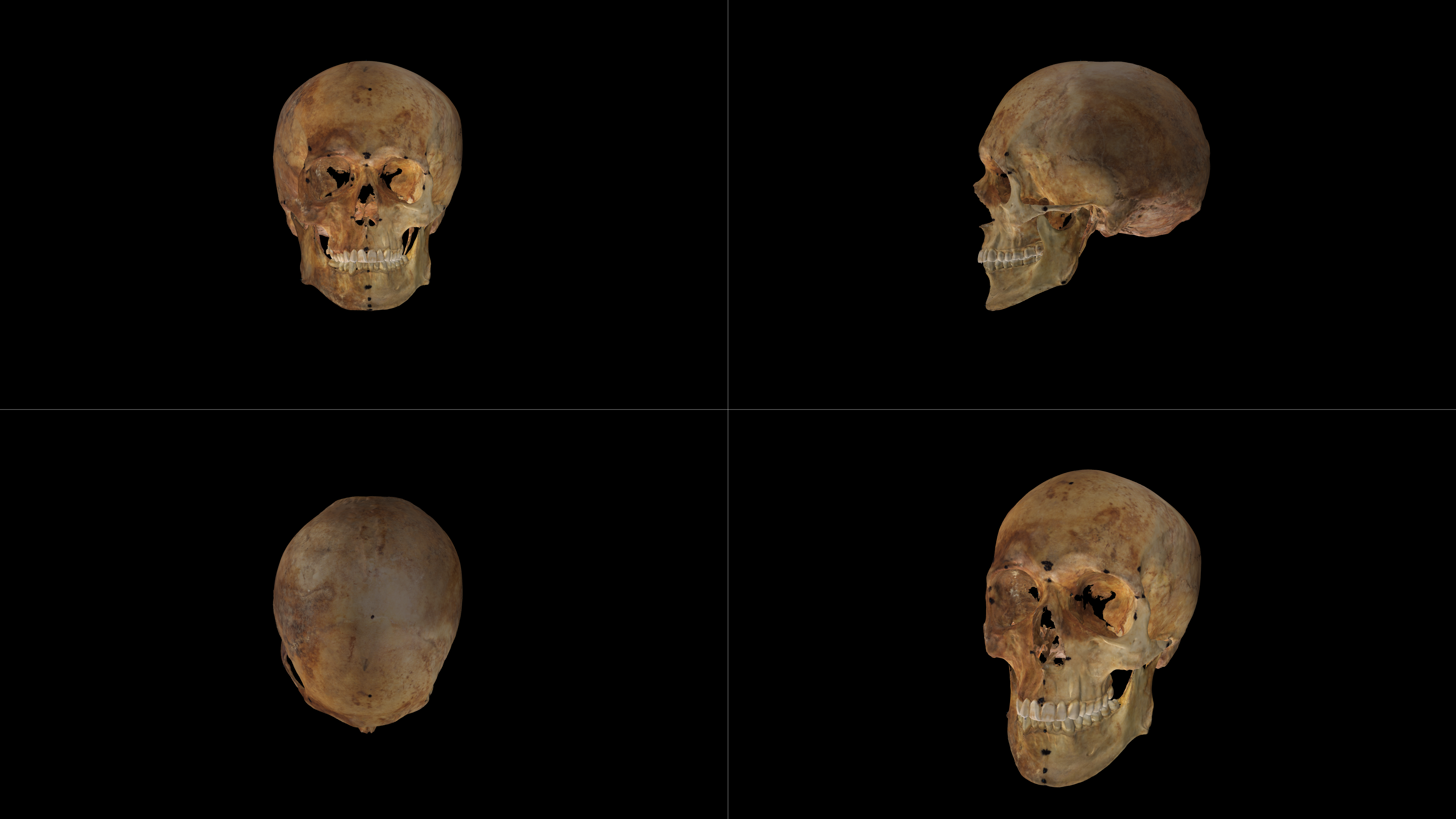

Supplement: Supplementary file 13 — Supplementary file13 (PNG 3012 KB) [file 414_2022_2929_MOESM13_ESM.png]

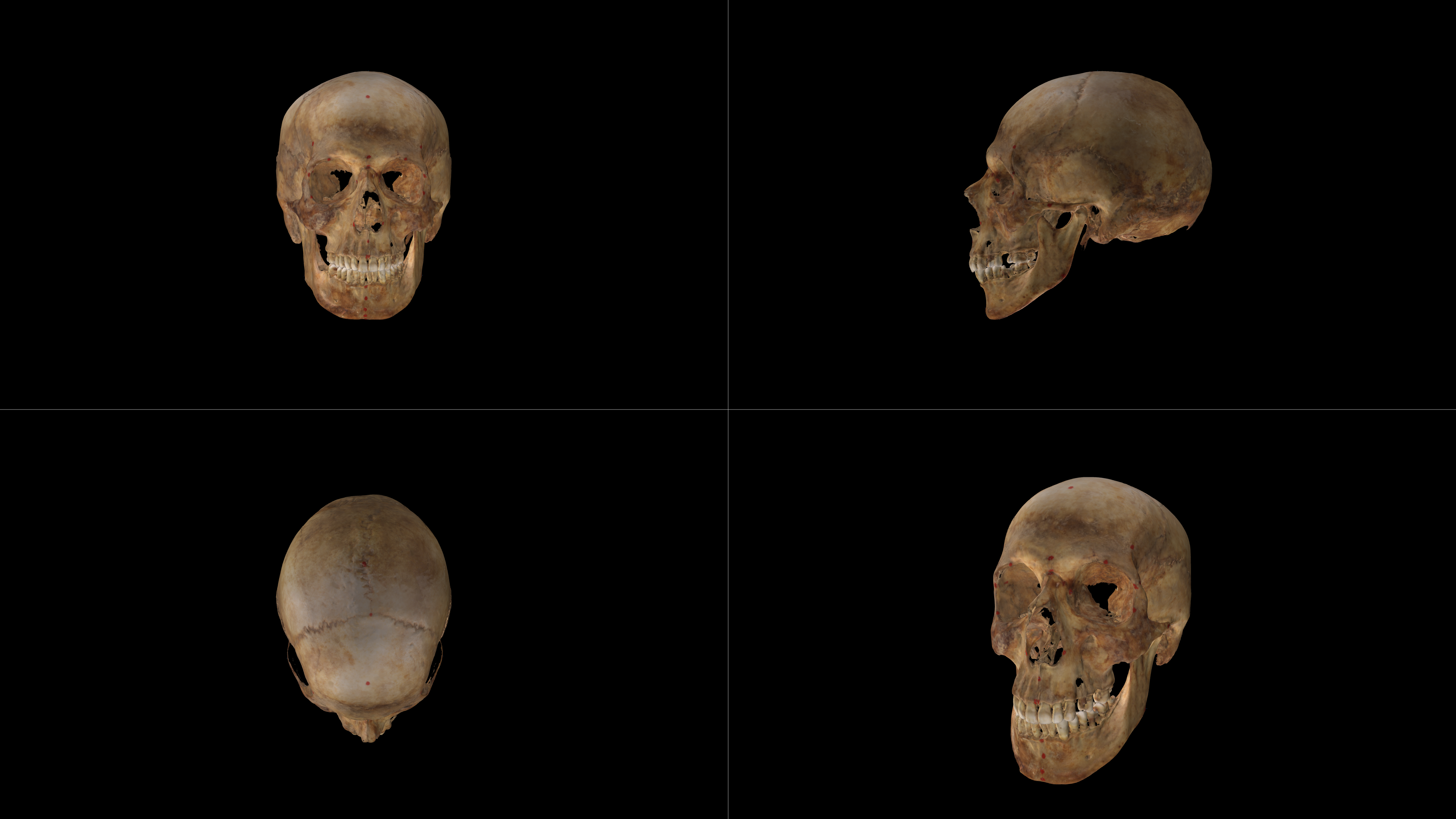

Supplement: Supplementary file 14 — Supplementary file14 (PNG 2761 KB) [file 414_2022_2929_MOESM14_ESM.png]

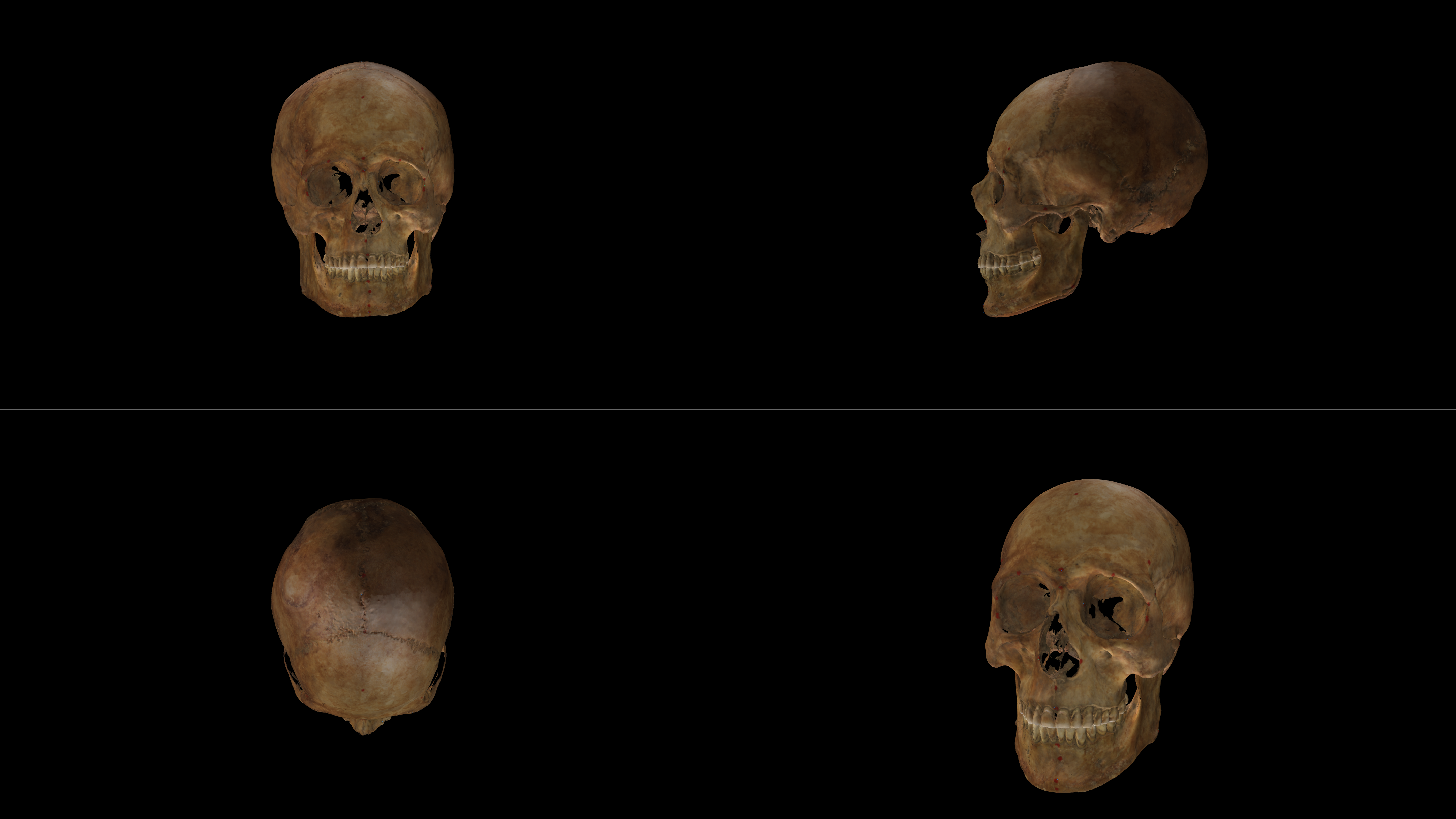

Supplement: Supplementary file 15 — Supplementary file15 (PNG 2700 KB) [file 414_2022_2929_MOESM15_ESM.png]

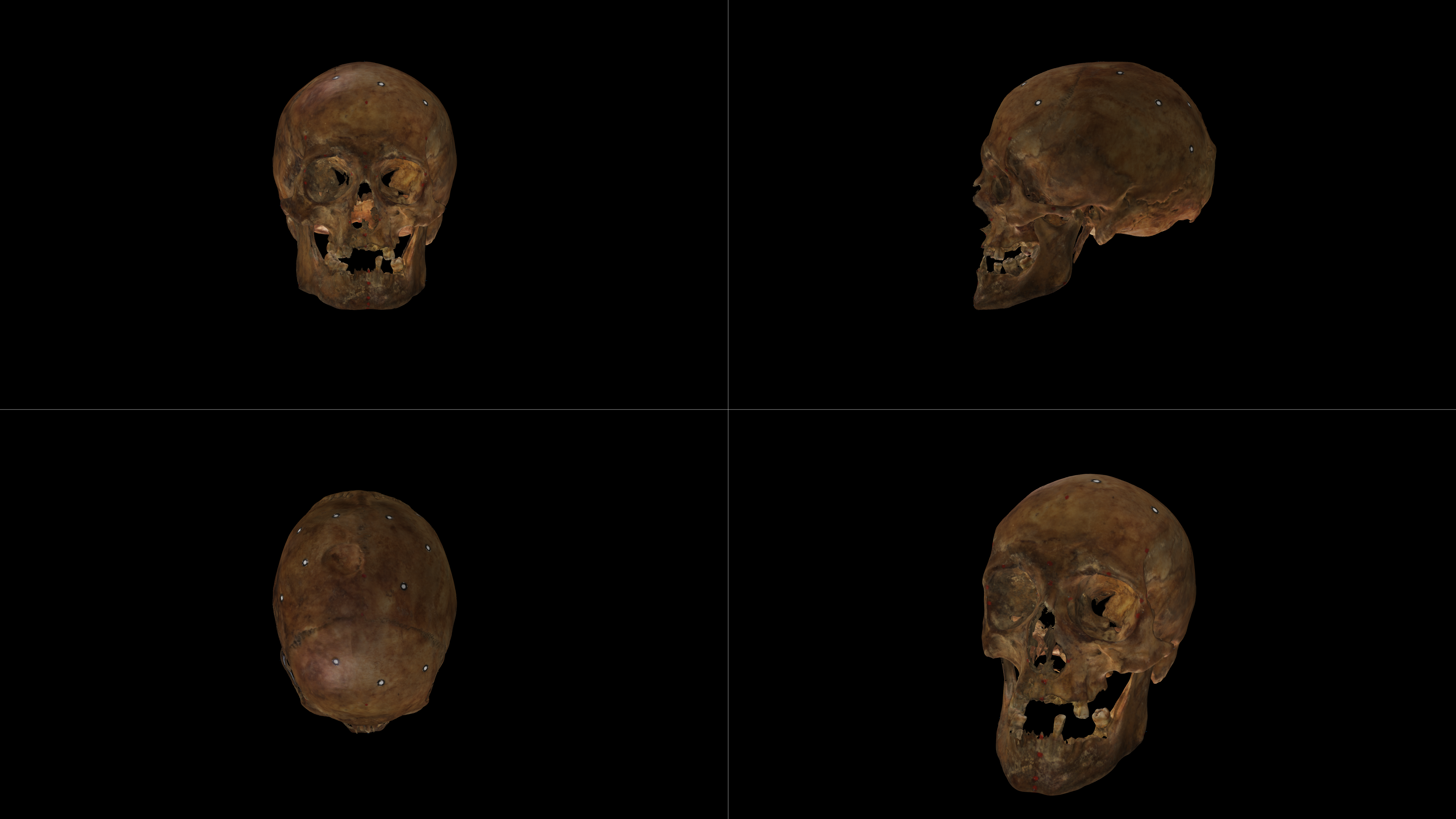

Supplement: Supplementary file 16 — Supplementary file16 (PNG 2699 KB) [file 414_2022_2929_MOESM16_ESM.png]

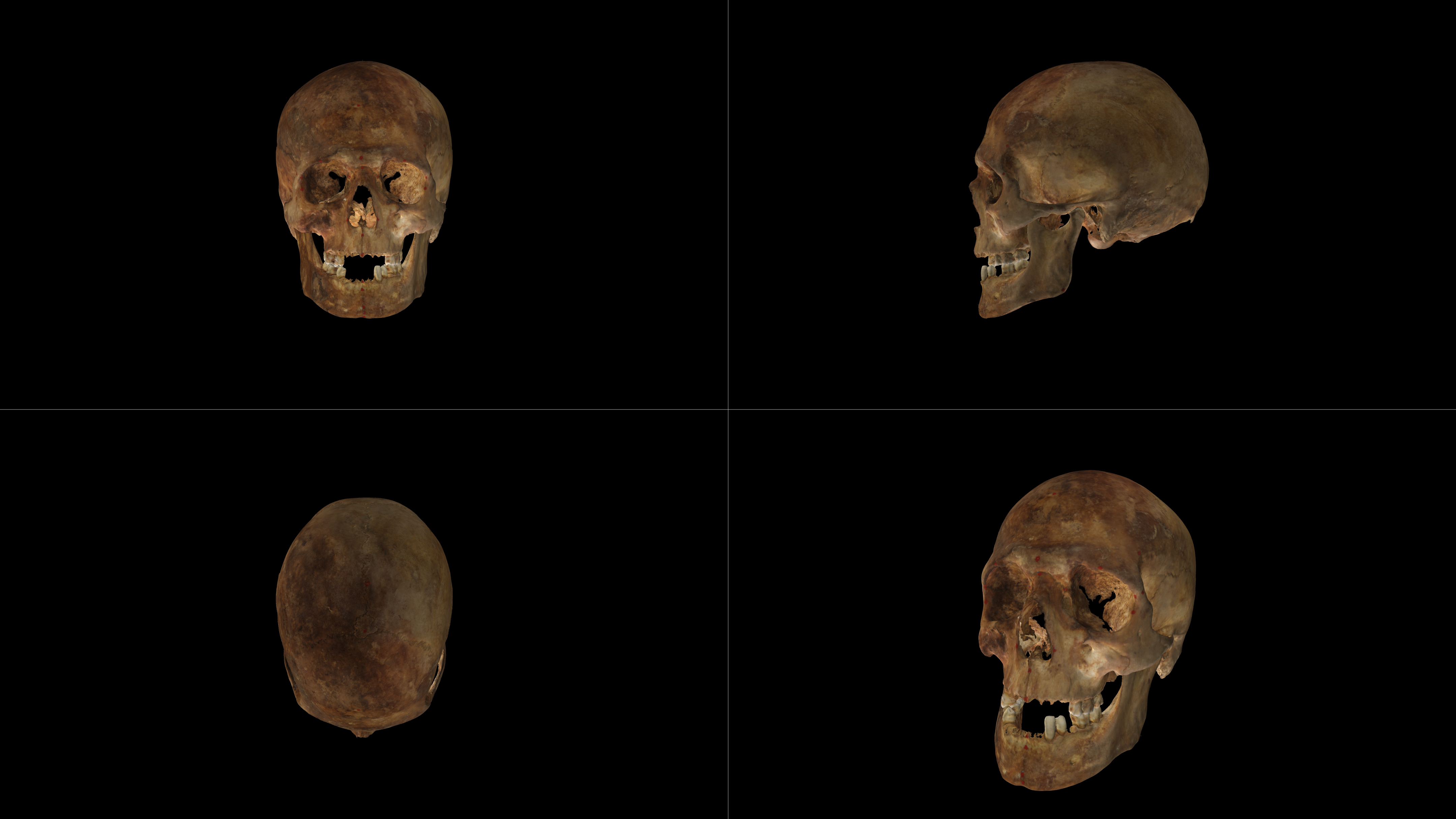

Supplement: Supplementary file 17 — Supplementary file17 (PNG 2902 KB) [file 414_2022_2929_MOESM17_ESM.png]

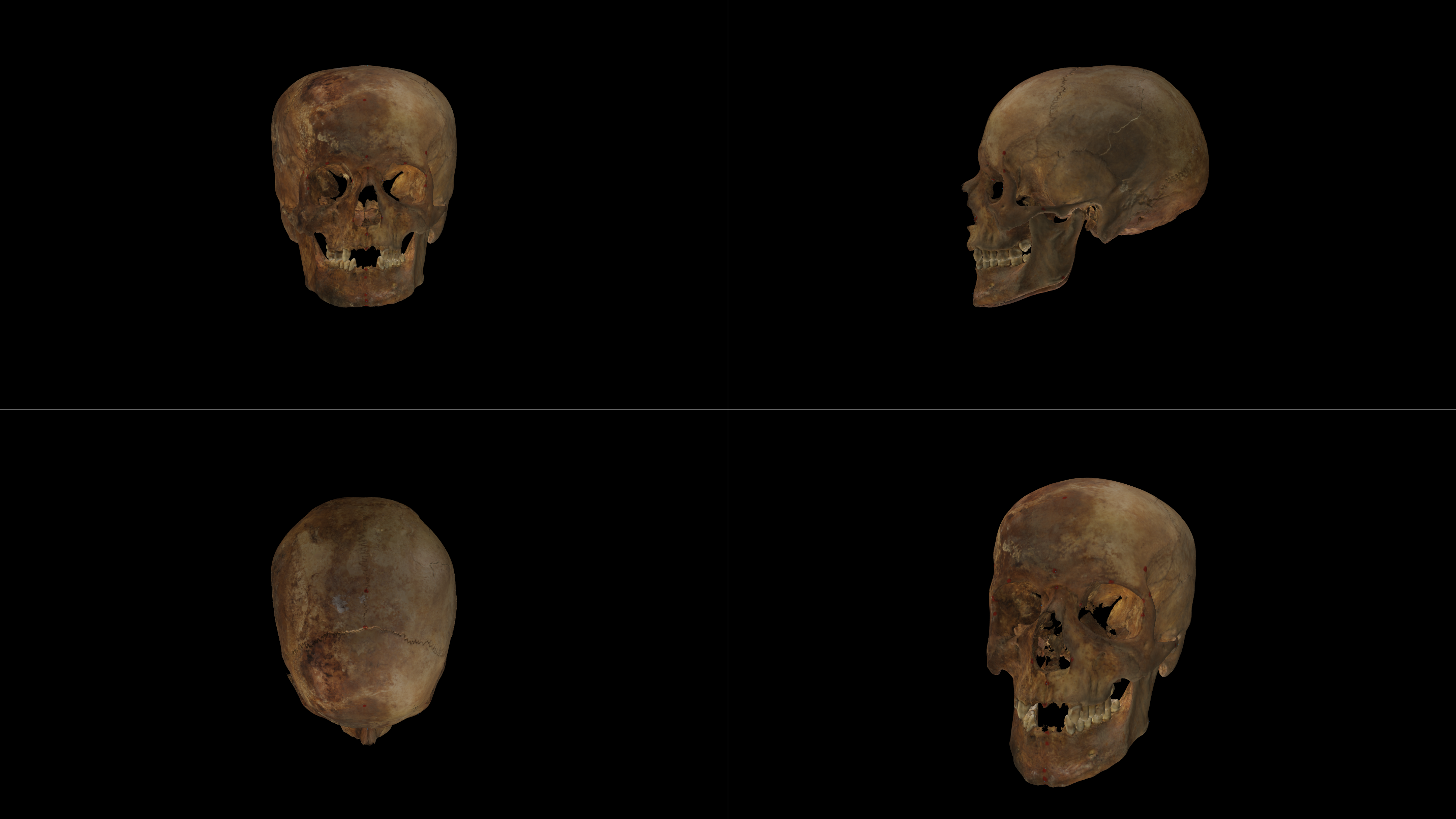

Supplement: Supplementary file 18 — Supplementary file18 (PNG 2823 KB) [file 414_2022_2929_MOESM18_ESM.png]
